# Supplementary material for: SLAPSHOT reveals rapid dynamics of extracellularly exposed proteome in response to calcium-activated plasma membrane phospholipid scrambling
Source: Commun Biol. 2024 Aug 29;7:1060. doi: 10.1038/s42003-024-06729-x (PMC11362511; doi:10.1038/s42003-024-06729-x)
Supplement: Supplementary file 2 — Supplementary Information [file 42003_2024_6729_MOESM2_ESM.pdf]

SLAPSHOT reveals rapid dynamics of extracellularly exposed proteome in response to calcium-activated plasma membrane phospholipid scrambling

Sami T. Tuomivaara*, Chin Fen Teo*, Yuh Nung Jan, Arun P. Wiita#, Lily Y. Jan#

Supplementary Figures

Figure **Page**

Supplementary Fig. 1……2

Supplementary Fig. 2……3

Supplementary Fig. 3……5

Supplementary Fig. 4……6

Supplementary Fig. 5……7

Supplementary Fig. 6……9

Supplementary Fig. 7……11

Supplementary Fig. 8……12

Supplementary Fig. 9……15

Supplementary Fig. 10……16

Supplementary Fig. 11……18

Supplementary Fig. 12……19

Supplementary Fig. 13……21

Supplementary Fig. 14……22

Supplementary Fig. 15……23

Supplementary Fig. 16……24

Supplementary Fig. 17……25

Supplementary Fig. 18……26

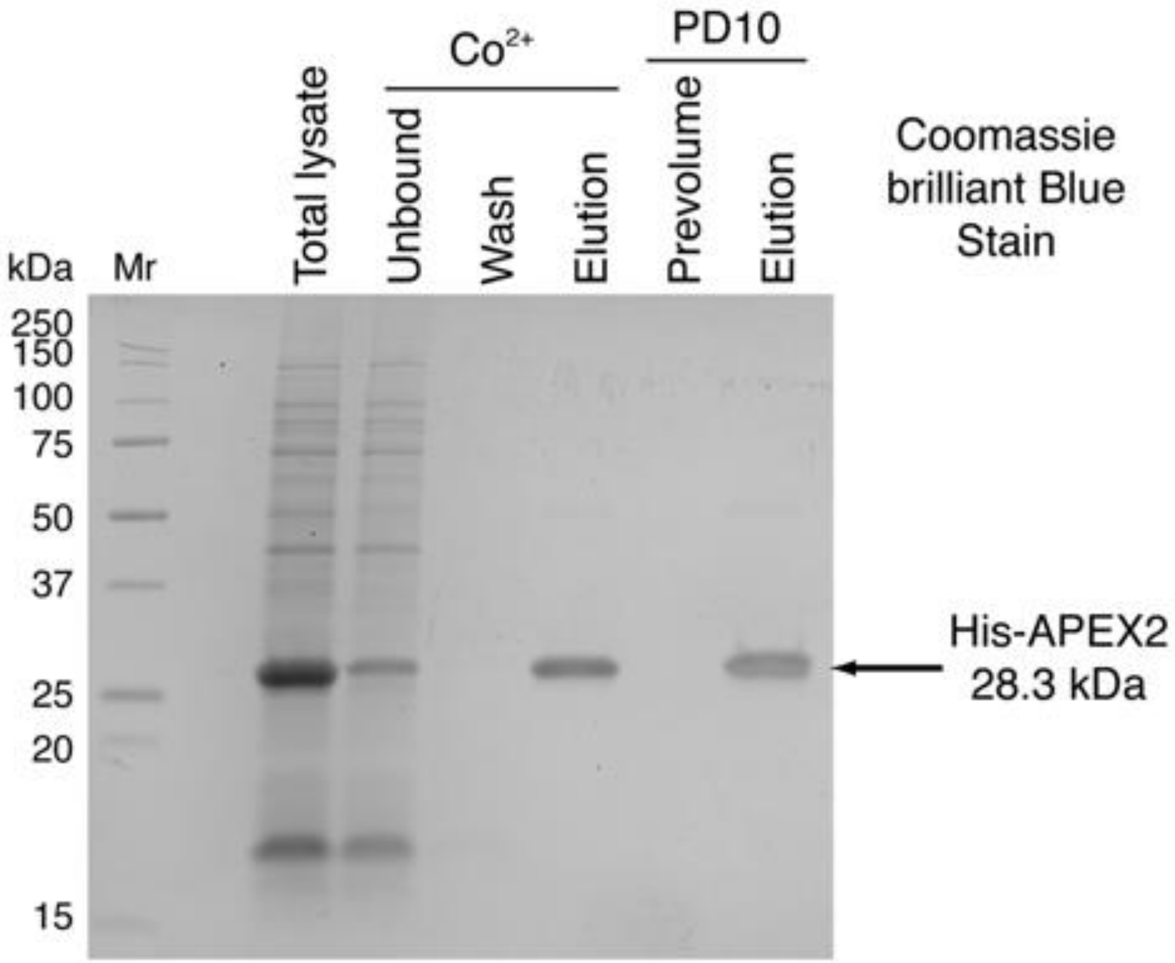

Supplementary Fig. 1 SDS-PAGE analysis of the purification of soluble recombinant His-APEX2 protein. Starting from a bacterial lysate, the purification workflow consists of cobalt-affinity chromatography, concentration using a spin filter, buffer exchange with PD10 column, heme reconstitution, excess heme removal, and a final spin filter concentration. The yields of APEX2 protein preparations are consistently >20 mg per 1 L of bacterial culture, and final densitometric purities >95%. Mr, relative molecular weight; kDa, kilodalton.

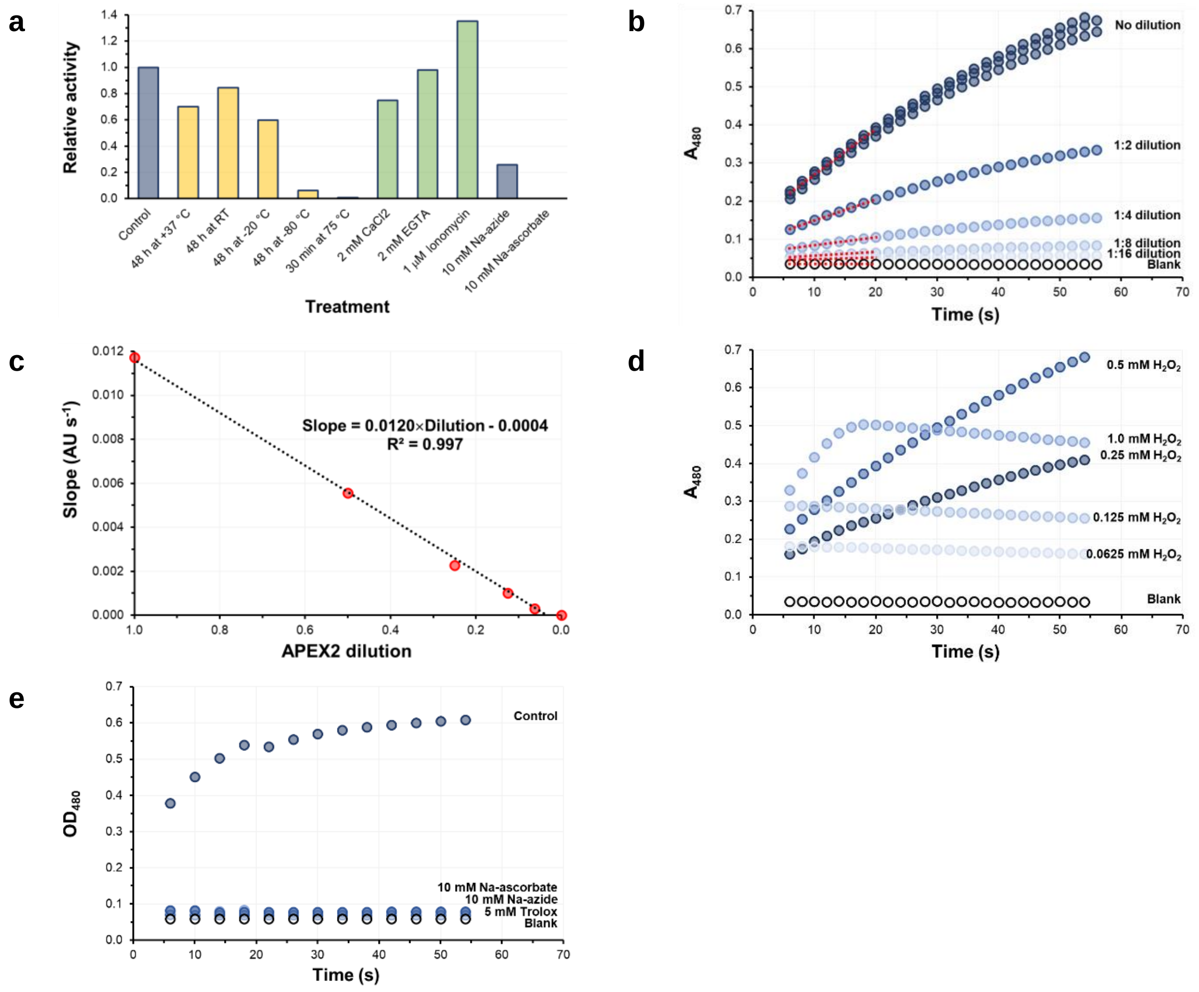

Supplementary Fig. 2 Effects of storage conditions, the concentration of reagents, and the dilution of APEX2 to the peroxidase activity. **a** Effect of the enzyme storage conditions, additives, and quenchers on APEX2 activity as per a colorimetric assay. The presented activities are reported relative to untreated freshly purified APEX2 enzyme kept at 4 °C. The data indicate that freezing either at -20 or -80 °C will reduce or completely abolish the APEX2 activity, we thus stored purified APEX2 proteins at 4 °C and freezing of APEX2 preparations was avoided altogether. 10 mM Na-azide (irreversible inhibitor) and 10 mM Na-ascorbate (reversible inhibitor) are effective inhibitors of the APEX2 enzyme. **b** Absorbance-vs-time curves from the colorimetric assay at various APEX2 dilutions. The APEX2 activity is equal to the initial (linear) slope of the absorbance-vs-time curve (*red dashed lines*). The high reproducibility of the assay is illustrated by the low coefficient of variation (< 3%) of triplicate measurements of the highest dilution used. **c** The initial slope of the absorbance-vs-time curve of the colorimetsric assay is linearly correlated with the used APEX2 dilution, indicating

the assay's suitability for gauging and normalizing APEX2 activities. **d** Effect of H_2O_2 concentration on APEX2 activity. The inhibitory effect of H_2O_2 is visible at concentrations higher than 0.5 mM. **e** Results from optical density-vs-time curves of turbidimetric assay utilizing the formation of colloid, presumably from cross-linked BT and APEX2, are in agreement with the colorimetric assay, in that 10 mM Na-azide and 10 mM Na-ascorbate are effective inhibitors of the APEX2 enzyme.

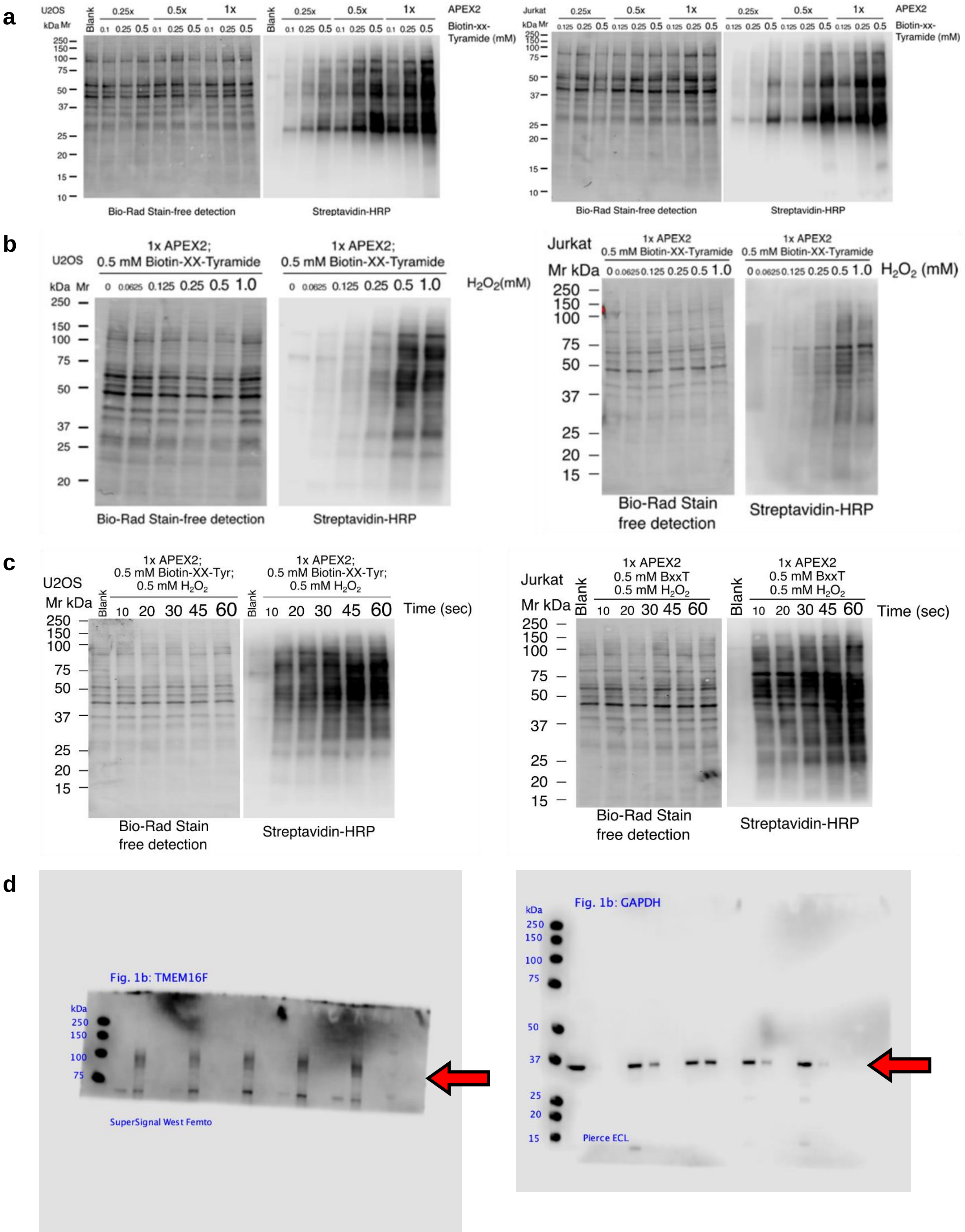
a
U2OS
kDa Mr
Blank
0.25x
0.5x
1x
APEX2
Biotin-xx-Tyramide (mM)
Bio-Rad Stain-free detection
Streptavidin-HRP
Jurkat
b
1x APEX2; 0.5 mM Biotin-XX-Tyramide
H2O2(mM)
Bio-Rad Stain-free detection
Streptavidin-HRP
Mr kDa
1x APEX2 0.5 mM Biotin-XX-Tyramide
H2O2 (mM)
Bio-Rad Stain free detection
c
1x APEX2; 0.5 mM Biotin-XX-Tyr; 0.5 mM H2O2
Time (sec)
1x APEX2 0.5 mM BxxT 0.5 mM H2O2
d
Fig. 1b: TMEM16F
SuperSignal West Femto
Fig. 1b: GAPDH
Pierce ECL

Supplementary Fig. 3 Optimization of SLAPSHOT labeling conditions. **a-c** Conditions were optimized separately for both adherent U2OS (*panel doublets on the left*) and suspended Jurkat (*panel doublets on the right*) cells. The optimized parameters included **a** APEX2 activity and BxxT concentration, **b** H_2O_2 concentration, **c** Labeling duration at room temperature. Each experiment includes the detection of total protein on the blotting membrane by stain-free imaging (*left panels*) and biotin incorporation by streptavidin-blotting (*right panels*). The data indicate that 30 or 45 s labeling respectively for adherent U2OS and suspended Jurkat cells using 0.0005 AU s^{-1} μL^{-1} (1× activity) (as measured by a colorimetric assay, see Supplementary Fig. 2a-c) and 0.5 mM BxxT and 0.5 mM H_2O_2 yield robust labeling. The H_2O_2 optimization corroborates the results obtained from streptavidin-blotting (Supplementary Fig. 2d). **d** Uncropped western blots of Fig 1b. Red arrow indicates the approximate molecular weight of the protein of interest. Mr, relative molecular weight; kDa, kilodalton.

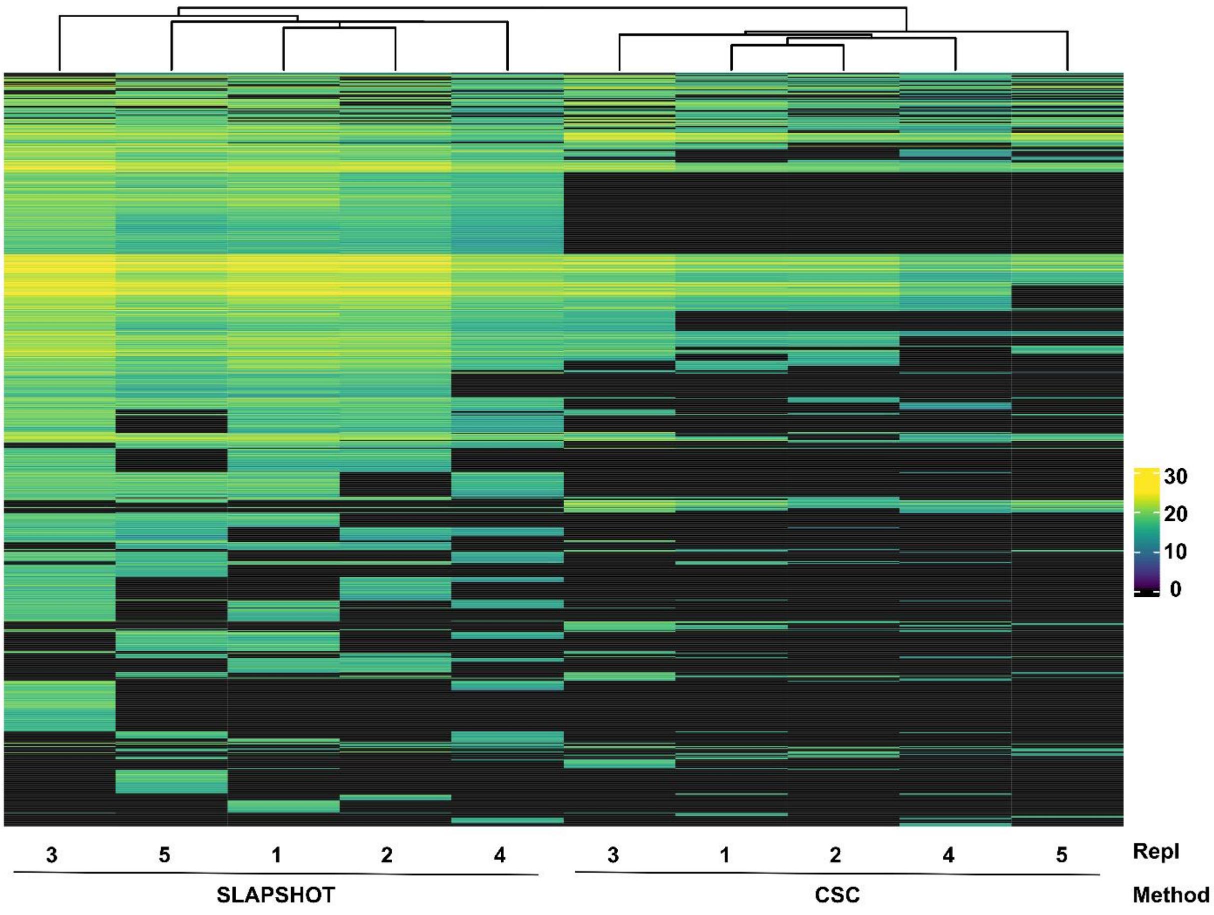

Supplementary Fig. 4 Hierarchical clustering of iBAQ intensities from U2OS cells labeled with SLAPSHOT or CSC. The iBAQ intensities cluster most strongly based on the method used, indicating that the methods label different subsets of proteins.

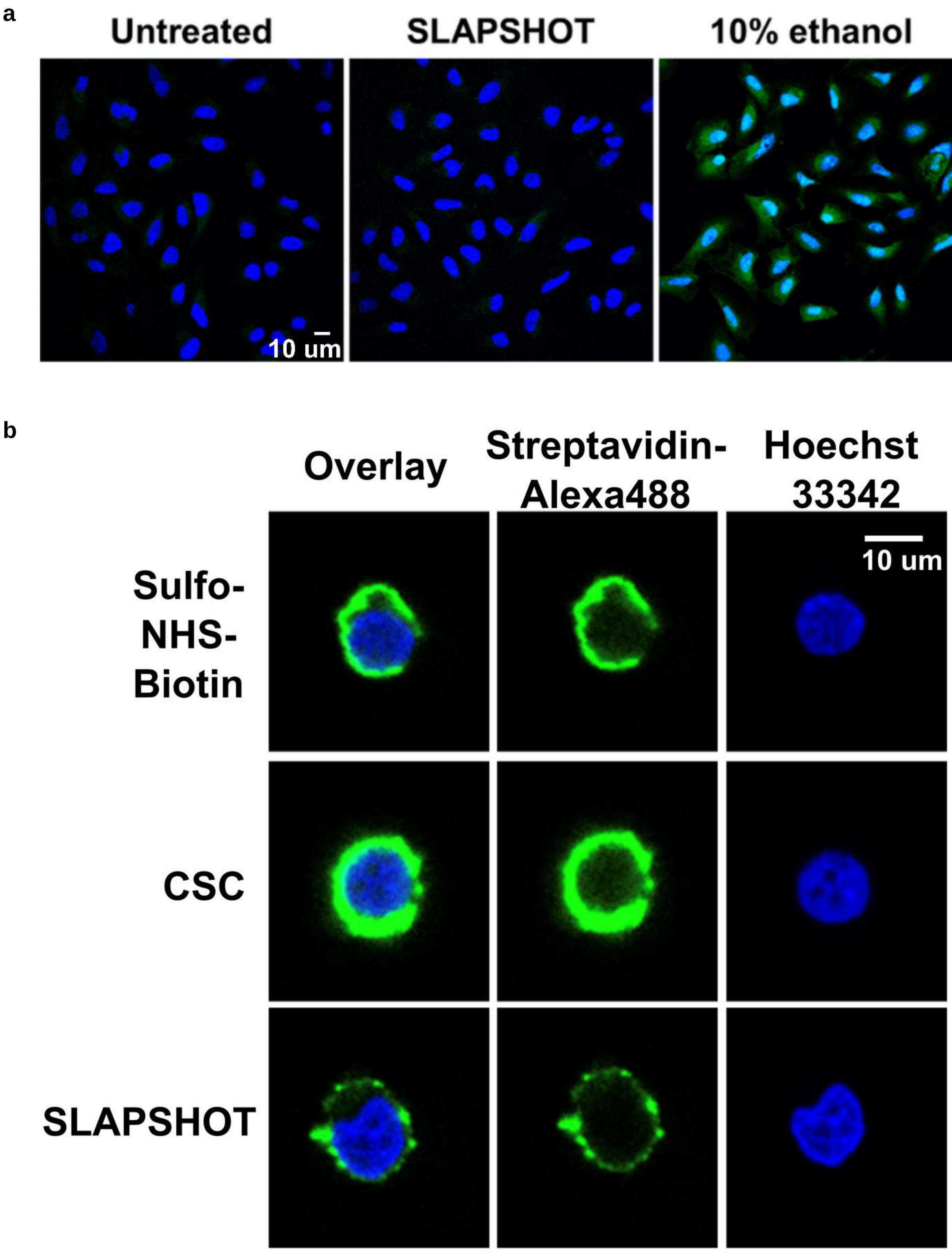
a
Untreated
SLAPSHOT
10% ethanol
10 um
b
Overlay
Streptavidin-Alexa488
Hoechst 33342
10 um
Sulfo-NHS-Biotin
CSC
SLAPSHOT

Supplementary Fig. 5 (*previous page*) SLAPSHOT preserves cellular integrity and selectively labels extracellularly exposed proteins. **a** Live-or-Dye assay on U2OS cells. The nuclei of all cells are labeled with Hoechst 33342 stain (*blue*), whereas Live-or-Dye reagent (*green*) only labels the cytoplasm of cells whose plasma membrane is compromised with 10% ethanol treatment. SLAPSHOT-treated cells are indistinguishable from untreated cells, indicating that SLAPSHOT does not compromise the integrity of the plasma membrane. **b** Microscopic examination of Jurkat cells after biotinylating the cells with either sulfo-NHS-biotin, CSC, or SLAPSHOT (all *green*). The nuclei of all cells are labeled with Hoechst 33342 stain (*blue*). The data indicate that the biotinylation is restricted to the cell periphery using all three methods.

a

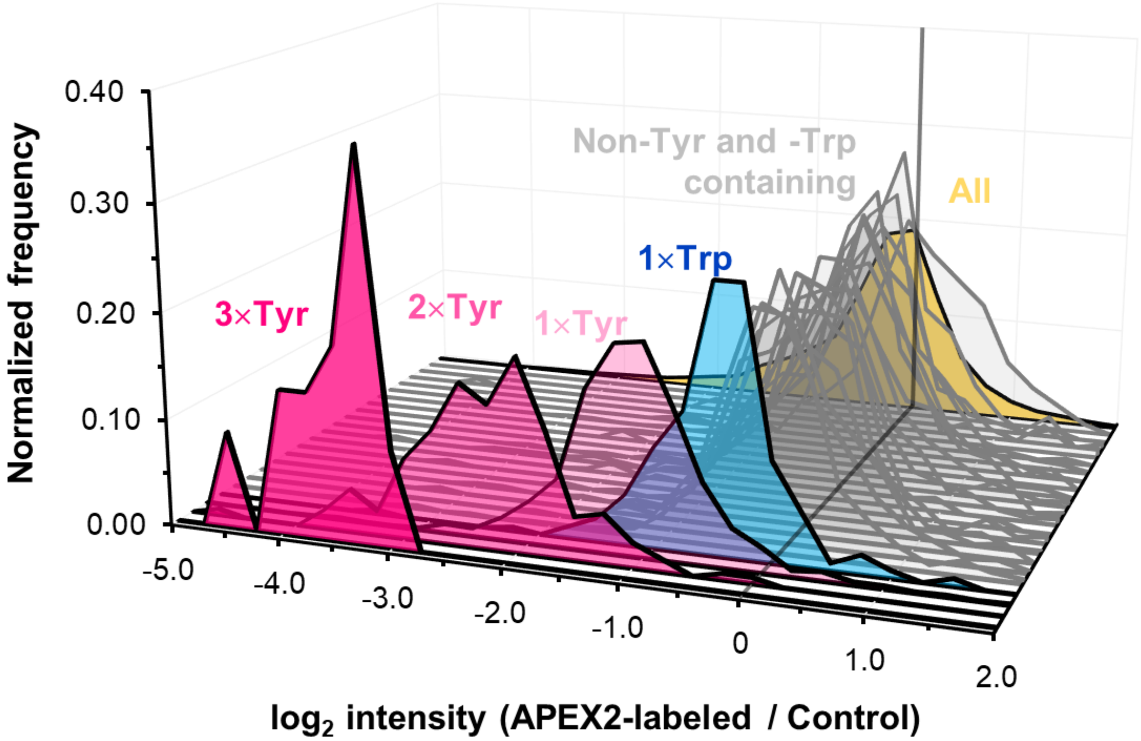

b

Peptides containing	N	Mean	SD	95% CI
**3 × Tyr**	22	-3.44	0.43	[-3.62 , -3.26]
**2 × Tyr**	171	-2.19	0.81	[-2.31 , -2.07]
**1 × Tyr**	1604	-0.99	0.68	[-1.02 , -0.95]
**1 × Trp**	275	-0.34	0.54	[-0.41 , -0.28]
**3 × Ala**	714	0.06	0.59	[0.02 , 0.11]
**3 × Arg**	121	0.49	0.59	[0.38 , 0.59]
**3 × Asn**	131	0.03	0.68	[-0.09 , 0.14]
**3 × Asp**	176	0.02	0.71	[-0.08 , 0.13]
**2 × Cys**	110	0.06	0.61	[-0.05 , 0.18]
**3 × Gln**	252	0.04	0.52	[-0.02 , 0.11]
**3 × Glu**	646	0.02	0.58	[-0.02 , 0.07]
**3 × Gly**	444	0.10	0.60	[0.04 , 0.15]
**2 × His**	109	0.29	0.78	[0.15 , 0.44]
**3 × Ile**	164	0.16	0.58	[0.07 , 0.24]
**3 × Leu**	596	0.13	0.60	[0.08 , 0.18]
**3 × Lys**	319	0.03	0.84	[-0.06 , 0.12]
**1 × Met**	496	0.05	0.67	[-0.01 , 0.11]
**2 × Phe**	319	0.06	0.57	[0.00 , 0.12]
**3 × Pro**	303	0.07	0.56	[0.01 , 0.14]
**3 × Ser**	400	0.11	0.67	[0.05 , 0.18]
**3 × Thr**	272	0.13	0.65	[0.05 , 0.20]
**3 × Val**	446	0.16	0.66	[0.10 , 0.22]
**Neither Tyr nor Trp**	7600	0.09	0.61	[0.07 , 0.10]
**Any amino acids**	9707	-0.16	0.81	[-0.17 , -0.14]

Only those Tyr-contaning pepides (*pink*) that do not contain Trp are considered.
Only those Trp-contaning peptides (*blue*) that do not contain Tyr are considered.
The rest of the analyzed peptides (grey) contain neither Tyr nor Trp.

Supplementary Fig. 6 *(previous page)* Phenoxyl radicals generated by APEX2 react solely with Tyr and Trp residues in proteins. Reduced and alkylated tryptic peptide mixture was treated with APEX2 in the presence of BP and H_2O_2, or left as an untreated control by omitting H_2O_2. After depleting the biotinylated materials from both reactions, supernatants were individually analyzed by MS in a data-dependent acquisition mode and MS^1-based quantitation. In order not to introduce any chance for target residue conflict between the quantitation reagent and the phenoxyl radicals, we performed label-free MS analyses. **a** To simplify the analysis, peptides quantified from only one of the samples were ignored, and $\log_2$ (APEX2-labeled/Control) intensity ratio was calculated for the rest of the peptides. When all of these peptides (N_{all} = 9707, *yellow*) are considered, the distribution of $\log_2$ ratios centers near zero, indicating globally similar peptide intensities in both samples. Only Tyr (*pink*) and Trp (*blue*) containing peptides have $\log_2$ intensity ratio distributions that are negatively shifted compared to the “All peptides” distribution, indicating their depletion from the APEX2-labeled mixture compared to the control mixture. In these calculations, only those Tyr-containing peptides that do not contain Trp, and those Trp-containing peptides that do not contain Tyr were included. Depletion efficiency increases linearly as the number of Tyr-residues in the peptide increases, as would be expected for independent phenoxyl radical targets. Due to the scarcity of Trp-containing residues, only those peptides with a single Trp were analyzed. These peptides exhibit depletion, albeit with lesser degree to those with a single Tyr residue, indicating lesser reactivity. The other eighteen traces (*gray*) represent peptides that do not contain Tyr or Trp residues but contain other residues of certain other type (typically 3×, depending on the amino acid abundance in the proteome, see b). Distributions of the $\log_2$ intensity ratios of these peptides center similarly to each other and to the “All peptides”, indicating that they are not effectively depleted from the biotinylated peptide mixture and thus none of the eighteen amino acids are targeted by the phenoxyl radicals. The Cys sulfhydryls were alkylated with iodoacetamide during peptide generation prior to APEX2 labeling, thus mimicking sulfhydryls in most extracellularly exposed proteins that are blocked by disulfide bonding. **b** Statistical analysis of the $\log_2$ (APEX2-labeled/Control) peptide intensity ratio distributions plotted in a. Shown are the number of peptides (*N*), mean, standard deviation (SD), and 95% confidence interval (CI) for the mean based on the normality assumption.

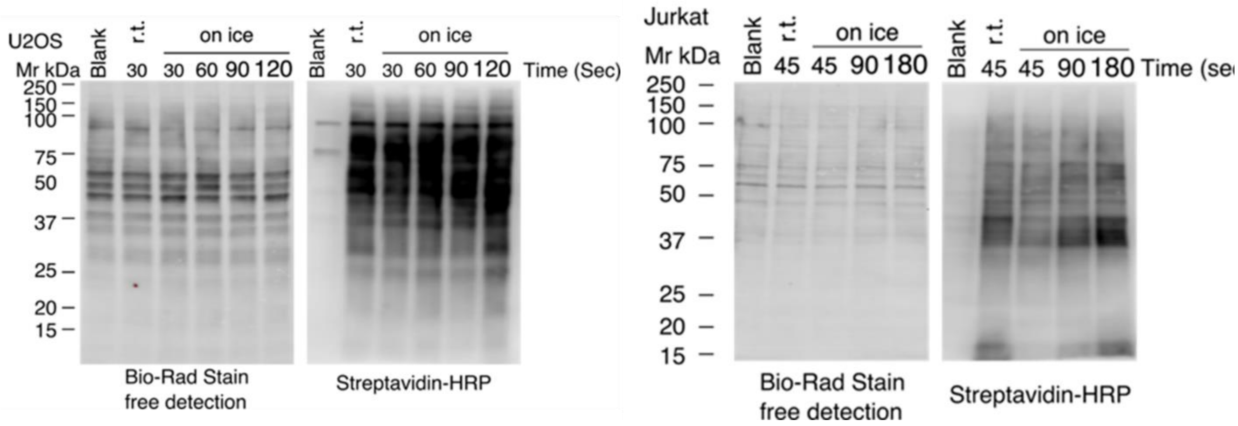

Supplementary Fig. 7 Optimization of SLAPSHOT labeling duration on ice. The labeling duration was optimized separately for both adherent U2OS (*panel doublet on the left*) and suspended Jurkat (*panel doublet on the right*) cells. Each experiment includes the detection of total protein on the blotting membrane by stain-free imaging (*left panels*) and biotin incorporation by Streptavidin-blotting (*right panels*). The data indicate that doubling the labeling time used for room temperature labeling, to 60 and 90 s for adherent U2OS and suspended Jurkat cells yields robust labeling. Mr, relative molecular weight; kDa, kilodalton.

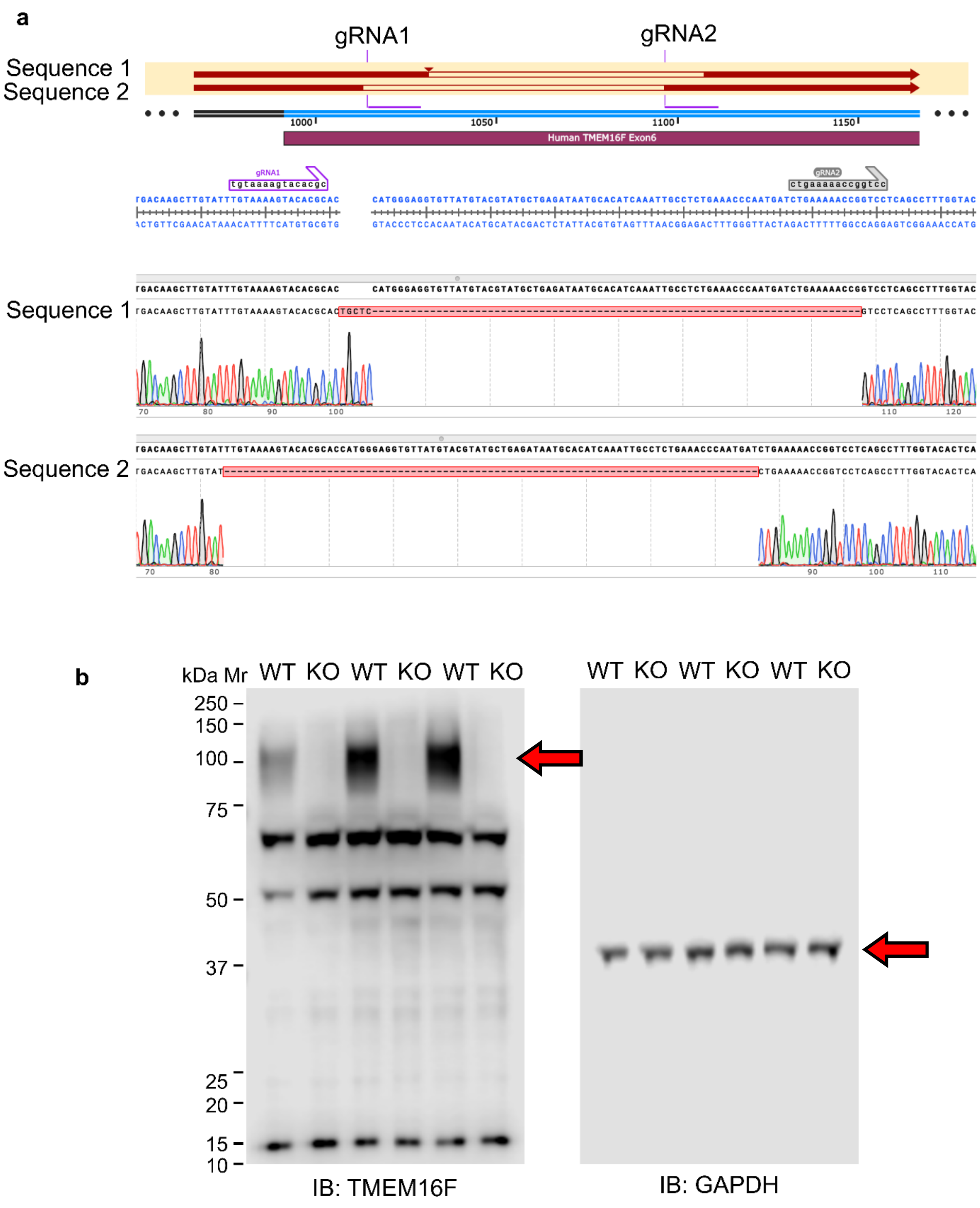

Supplementary Fig. 8 Genetic and biochemical verification of *ANO6* CRISPR-Cas9 knock-out in Jurkat cells. **a** Sanger sequencing results indicate that the knock-out cell population is either a mixture of two clones, both with homozygous deletions in the exon 6 of

ANO6, or a single heterozygous clone. The deletions lead to frameshifts and premature stop codons that create truncated transcripts. **b** Uncropped western blots of three individually expanded clones of both WT and 16FKO cells indicates the absence of TMEM16F protein in the knock-out cells, whereas control GAPDH levels are comparable in the two cell types. Red arrow indicates the approximate molecular weight of the protein of interest. Mr, relative molecular weight; kDa, kilodalton.

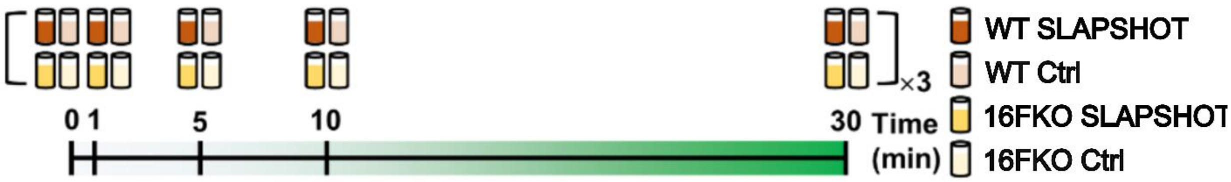

Supplementary Fig. 9 Schematic of the time-course ionomycin stimulation of Jurkat cells. WT and 16FKO cells were stimulated with bacterial calcium ionophore ionomycin for durations ranging from 1 to 30 min. Cells at resting state that were not stimulated (0 min) were included as well. The samples were labeled with SLAPSHOT according to the optimized protocol. Matching control without H_2O_2 was included for each individual labeled sample. The experiment was replicated three times using different passages of Jurkat cells.

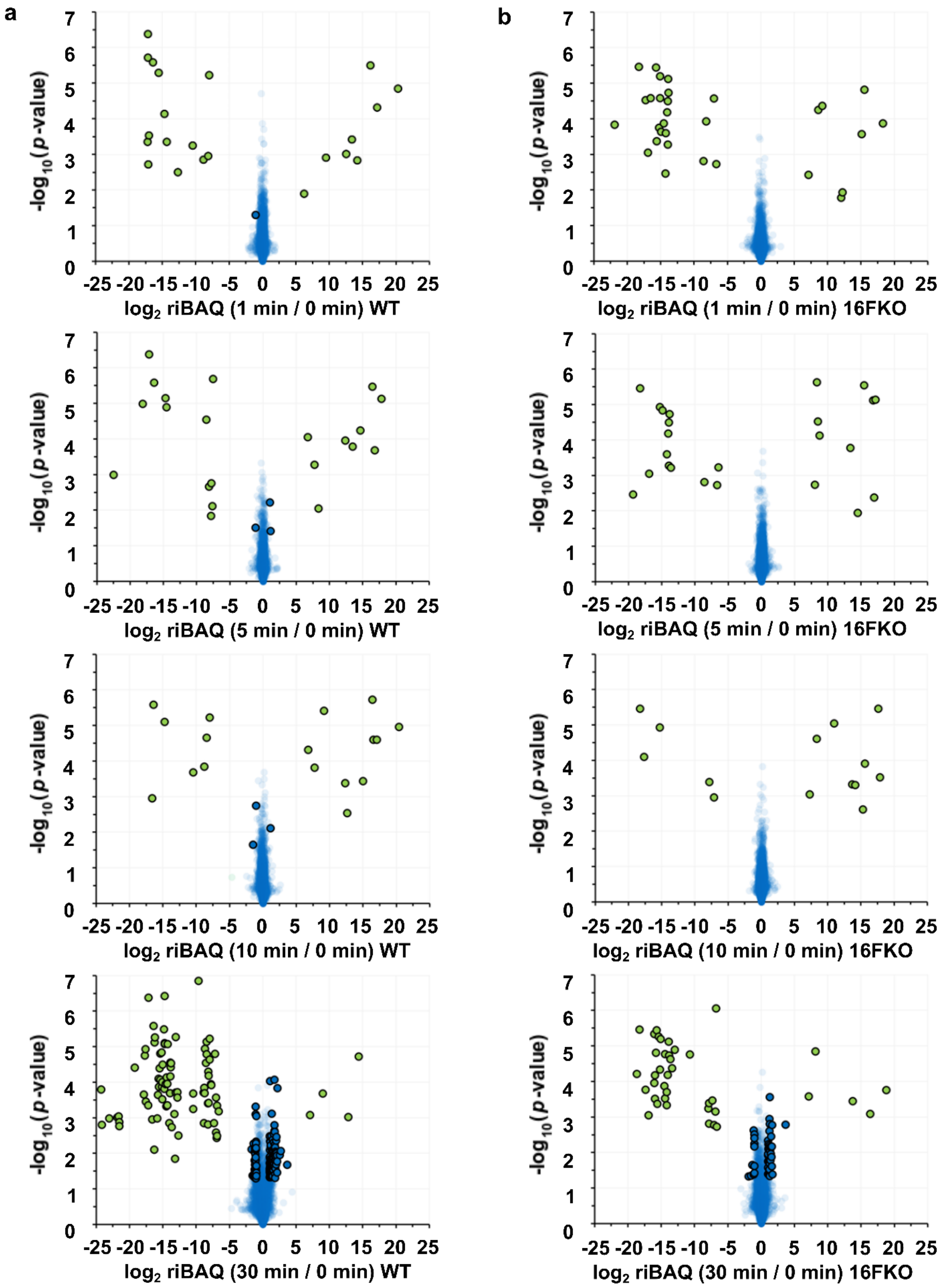
a
b
-log10(p-value)
log2 riBAQ (1 min / 0 min) WT
log2 riBAQ (1 min / 0 min) 16FKO
log2 riBAQ (5 min / 0 min) WT
log2 riBAQ (5 min / 0 min) 16FKO
log2 riBAQ (10 min / 0 min) WT
log2 riBAQ (10 min / 0 min) 16FKO
log2 riBAQ (30 min / 0 min) WT
log2 riBAQ (30 min / 0 min) 16FKO

Supplementary Fig. 10 ***(previous page)*** **Volcano plots indicating the proteome changes in control and variously ionomycin treated cells.** **a** WT and **b** 16FKO Jurkat cells. Datapoints in *blue* indicate proteins that were detected in both samples, whereas data points in *green* indicate proteins that were identified in just one sample and imputed in the other (the *green* data points are included merely for visualization purposes). Proteins whose absolute $\log_2$ riBAQ fold-change >1, and $-\log_{10}$ *p*-value >1.3 (*p*-value < 0.05) are *circled*. The data indicate that a relatively small number of proteins are differentially detected between 0 min and any of the shorter stimulation endpoints (1, 5, or 10 min) in both WT and 16FKO cells, whereas a larger number of proteins are differentially detected between 0 min and 30 min stimulation in both cell types.

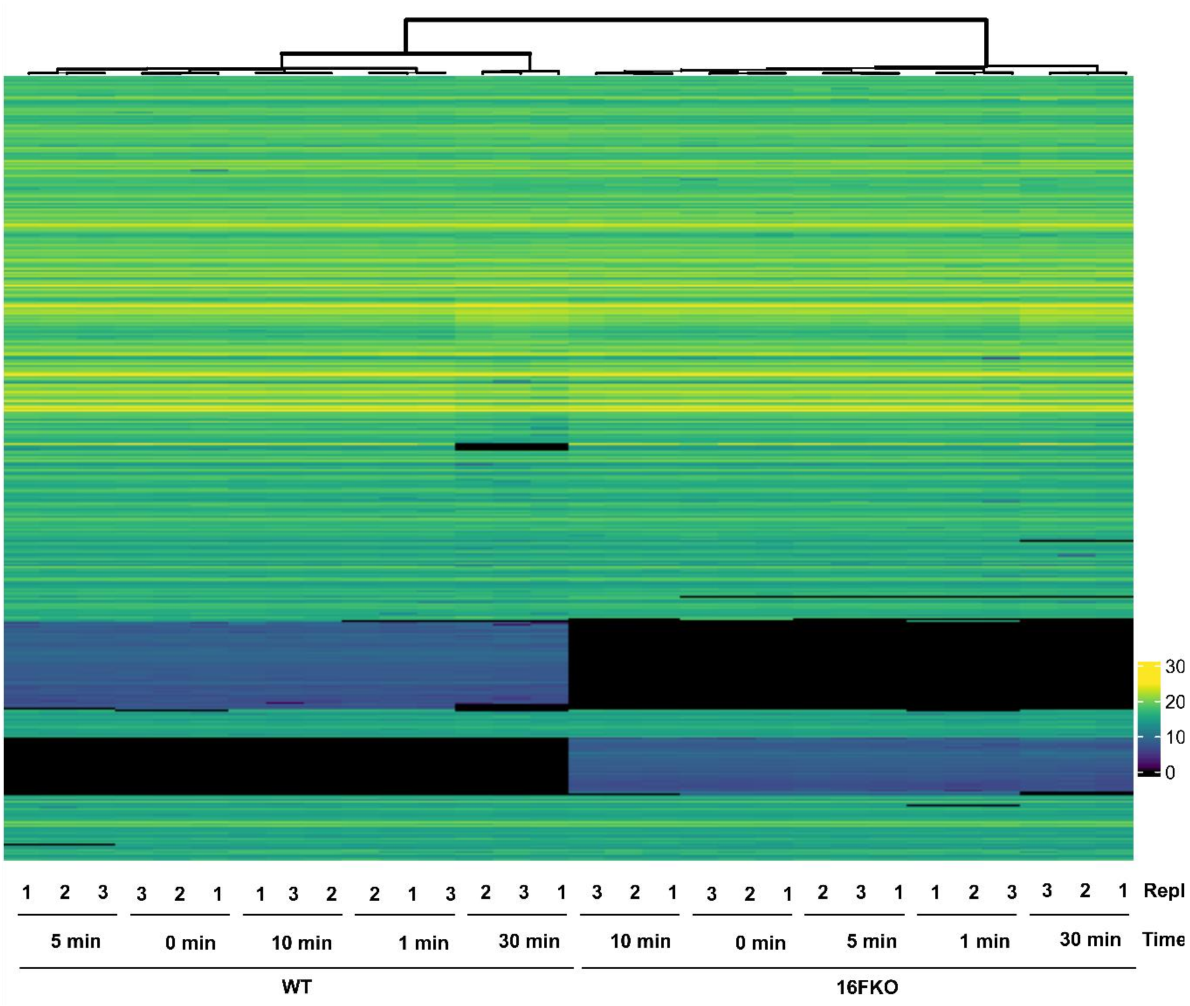

Supplementary Fig. 11 Hierarchical clustering of WT and 16FKO Jurkat cell ionomycin stimulation time-course data. The TMT-riBAQ intensities separate strongly based on the cell type, followed by time point. The data indicates strong reproducibility of the triplicate measurements from a given cell type and time point.

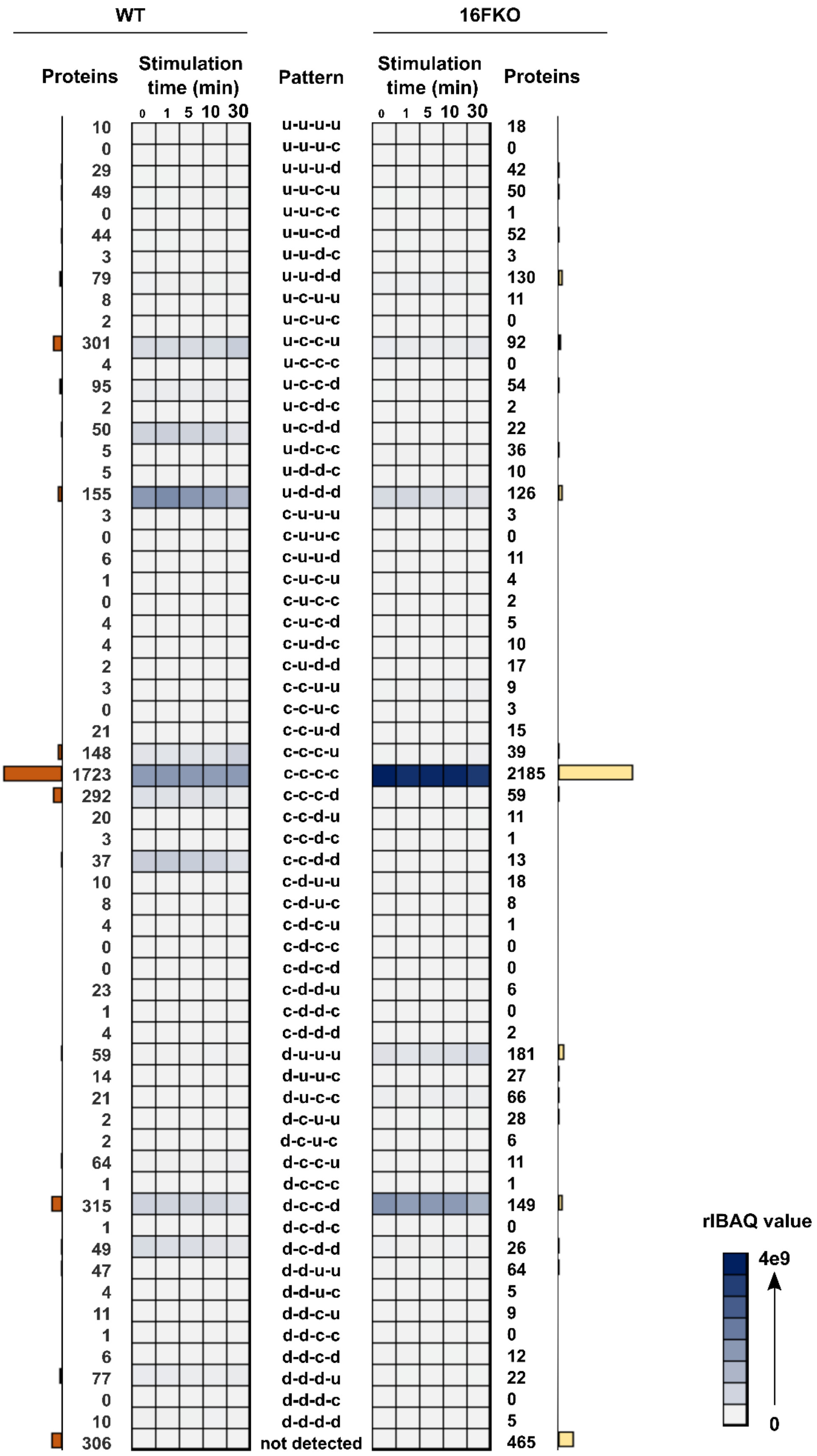
WT
16FKO
Proteins
Stimulation time (min)
0 1 5 10 30
Pattern
Stimulation time (min)
0 1 5 10 30
Proteins
10 u-u-u-u 18
0 u-u-u-c 0
29 u-u-u-d 42
49 u-u-c-u 50
0 u-u-c-c 1
44 u-u-c-d 52
3 u-u-d-c 3
79 u-u-d-d 130
8 u-c-u-u 11
2 u-c-u-c 0
301 u-c-c-u 92
4 u-c-c-c 0
95 u-c-c-d 54
2 u-c-d-c 2
50 u-c-d-d 22
5 u-d-c-c 36
5 u-d-d-c 10
155 u-d-d-d 126
3 c-u-u-u 3
0 c-u-u-c 0
6 c-u-u-d 11
1 c-u-c-u 4
0 c-u-c-c 2
4 c-u-c-d 5
4 c-u-d-c 10
2 c-u-d-d 17
3 c-c-u-u 9
0 c-c-u-c 3
21 c-c-u-d 15
148 c-c-c-u 39
1723 c-c-c-c 2185
292 c-c-c-d 59
20 c-c-d-u 11
3 c-c-d-c 1
37 c-c-d-d 13
10 c-d-u-u 18
8 c-d-u-c 8
4 c-d-c-u 1
0 c-d-c-c 0
0 c-d-c-d 0
23 c-d-d-u 6
1 c-d-d-c 0
4 c-d-d-d 2
59 d-u-u-u 181
14 d-u-u-c 27
21 d-u-c-c 66
2 d-c-u-u 28
2 d-c-u-c 6
64 d-c-c-u 11
1 d-c-c-c 1
315 d-c-c-d 149
1 d-c-d-c 0
49 d-c-d-d 26
47 d-d-u-u 64
4 d-d-u-c 5
11 d-d-c-u 9
1 d-d-c-c 0
6 d-d-c-d 12
77 d-d-d-u 22
0 d-d-d-c 0
10 d-d-d-d 5
306 not detected 465
rIBAQ value
4e9
0

Supplementary Fig. 12 *(previous page)* Heatmaps showing the evolution of the total riBAQ for intensity patterns. Data are shown for WT (*left heatmap*) and 16FKO (*right heatmap*) cells. All detected proteins (N = 4148) are considered. Each square in the grid represents the sum of riBAQ values from the proteins with that intensity pattern. The total number of proteins belonging to a pattern are indicated by numbers as well bar graphs. Notably, most of the proteins and riBAQ intensity are concentrated in a few patterns.

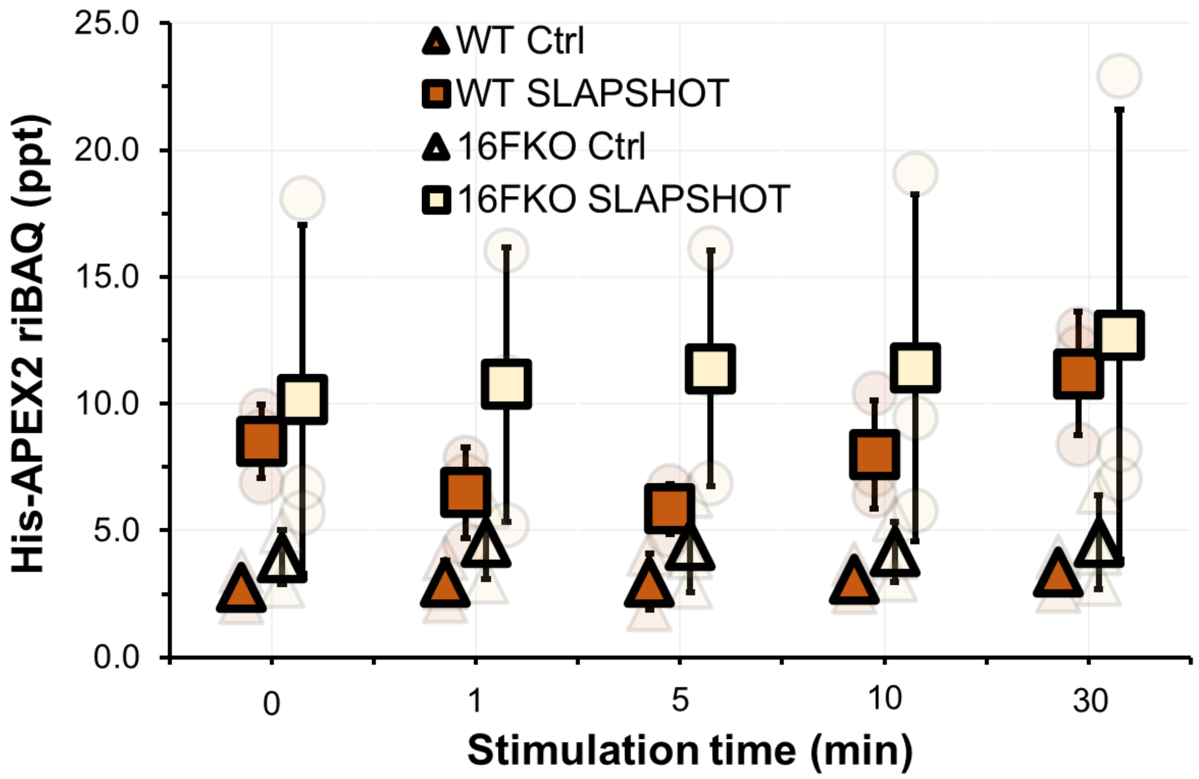

Supplementary Fig. 13 His-APEX2 protein riBAQ intensities in control and labeled samples from WT and 16FKO cells. Mean (*dark fills*) ± standard deviation indicated, *N* = 3 (individual datapoints in *light fills*). The data indicate that all cell pellets from the labeling reactions consistently contain ~2.5-fold more APEX2 protein compared to their corresponding non-labeled control cell pellets. This low and consistent ratio indicates that there is no major cellular internalization of the His-APEX2 protein during the labeling reaction, either by calcium-dependent (*e.g.*, endocytosis) or other processes. Rather, non-specific binding of a colloid formed by cross-linked APEX2 and BxxT to cells, plasticware, and Neutravidin-agarose contribute to the His-APEX2 riBAQ intensity in the samples. ppt, parts per thousand.

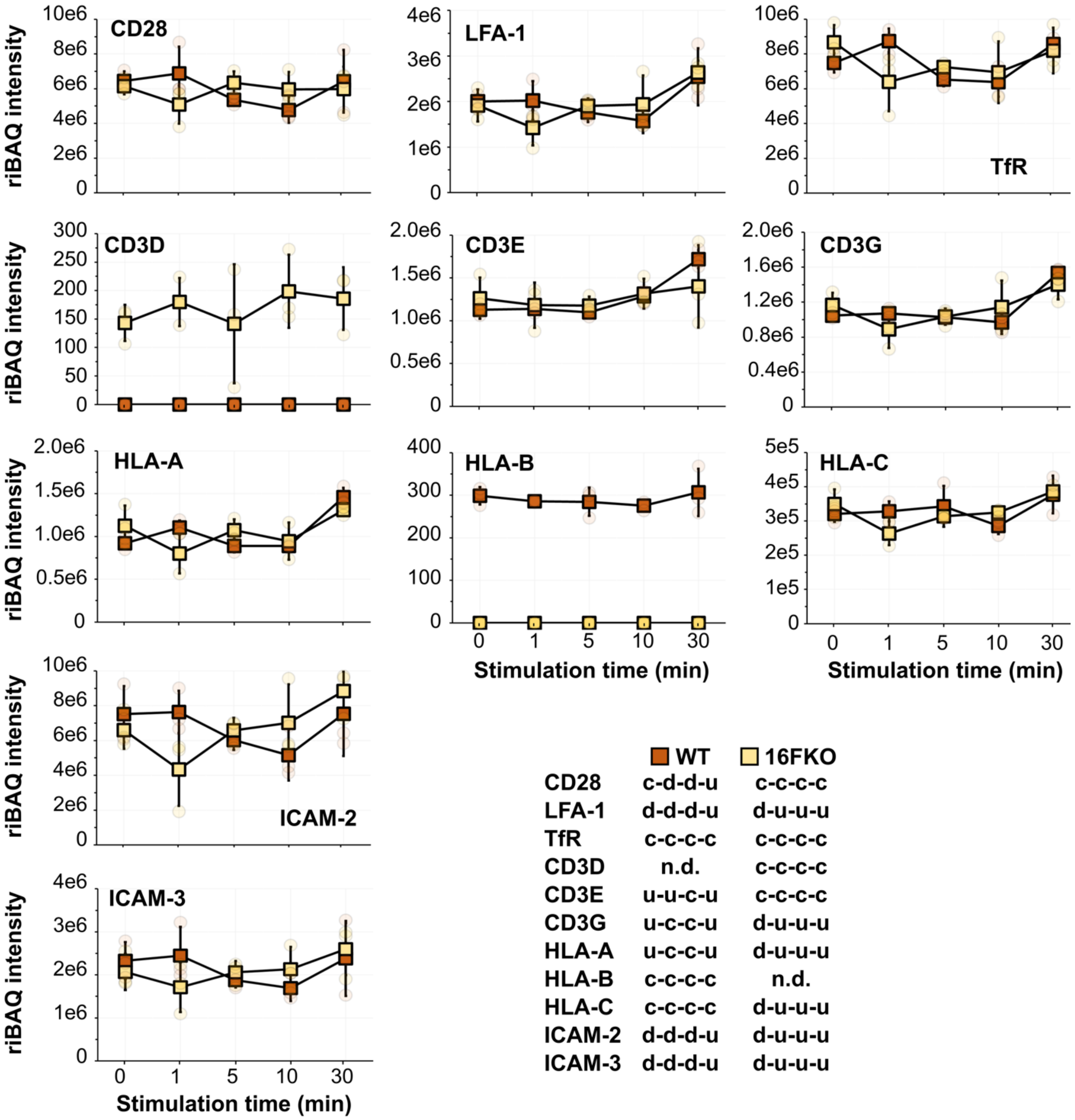

	WT	16FKO
CD28	c-d-d-u	c-c-c-c
LFA-1	d-d-d-u	d-u-u-u
TfR	c-c-c-c	c-c-c-c
CD3D	n.d.	c-c-c-c
CD3E	u-u-c-u	c-c-c-c
CD3G	u-c-c-u	d-u-u-u
HLA-A	u-c-c-u	d-u-u-u
HLA-B	c-c-c-c	n.d.
HLA-C	c-c-c-c	d-u-u-u
ICAM-2	d-d-d-u	d-u-u-u
ICAM-3	d-d-d-u	d-u-u-u

Supplementary Fig. 14 MS time-course profiles for selected proteins. Mean (*dark fills*) ± standard deviation indicated, $N = 3$ (individual datapoints in *light fills*). These proteins were monitored by Bricogne *et al.* (2019), using PD-1 overexpressing Jurkat cells with 15 min of 5 µM ionomycin stimulation, or proteins in the same family. n.d., not detected.

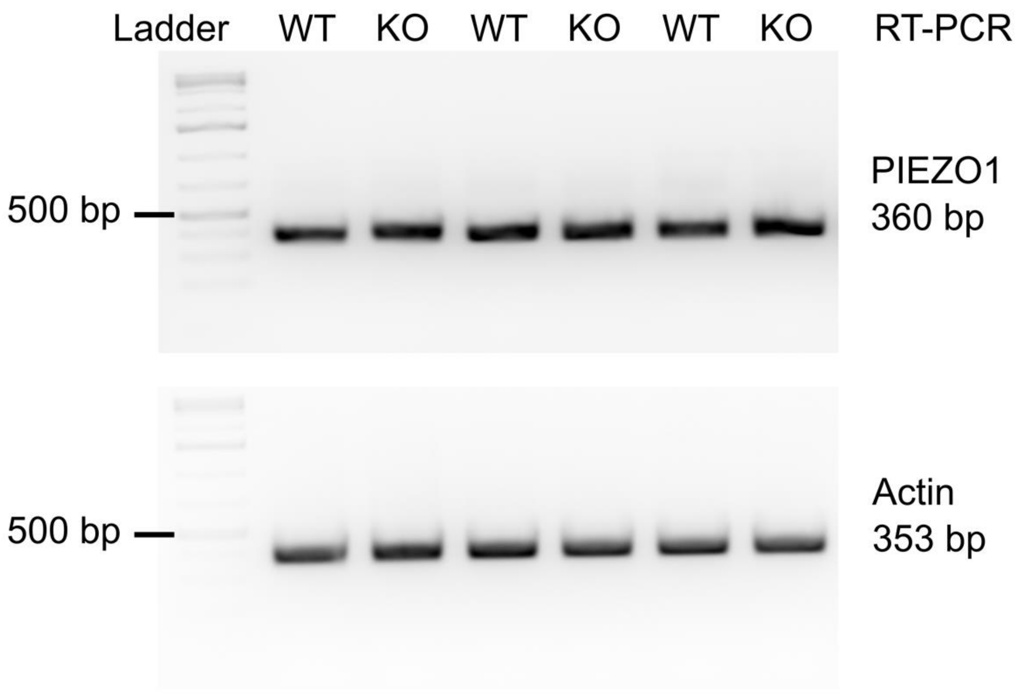

Supplementary Fig 15 RT-PCR analysis of PIEZO1. PIEZO1 (*upper panel*) and actin (*lower panel*) transcripts are shown for WT and 16FKO Jurkat cells. Data from three replicates indicates the presence of PIEZO1 transcript in the 16FKO cells at comparable levels to that in the WT cells. bp, base pairs.

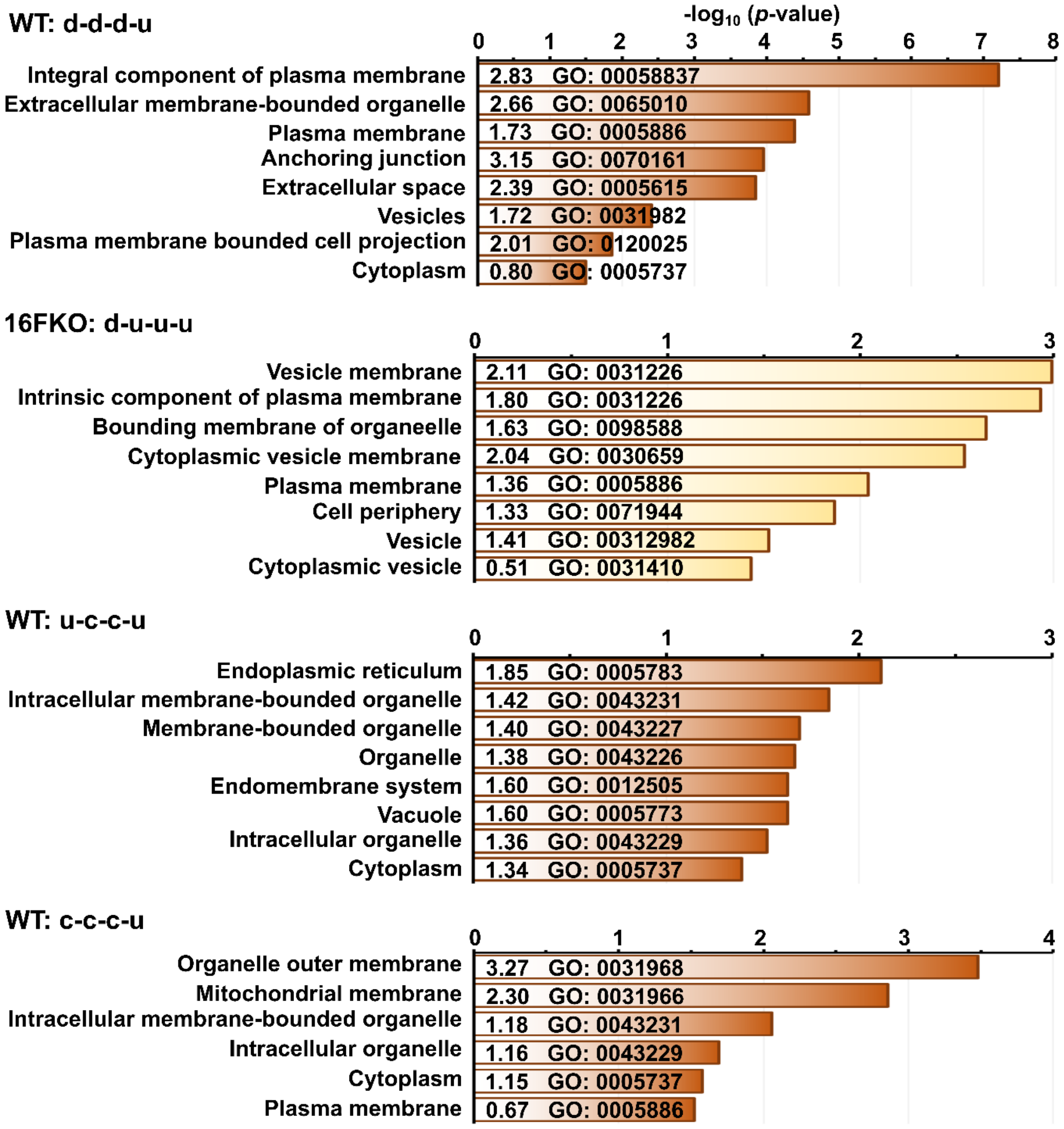

Supplementary Fig. 16 PANTHER analysis (GO cellular component) of proteins with selected intensity patterns. The length of the bar indicates significance with $-\log_{10}$ (*p*-value). GO terms and descriptions are shown, as are the enrichment factors. Enrichment factors > 1 indicate overrepresentation of proteins in that category in the data, whereas enrichment factor < 1 indicates depletion.

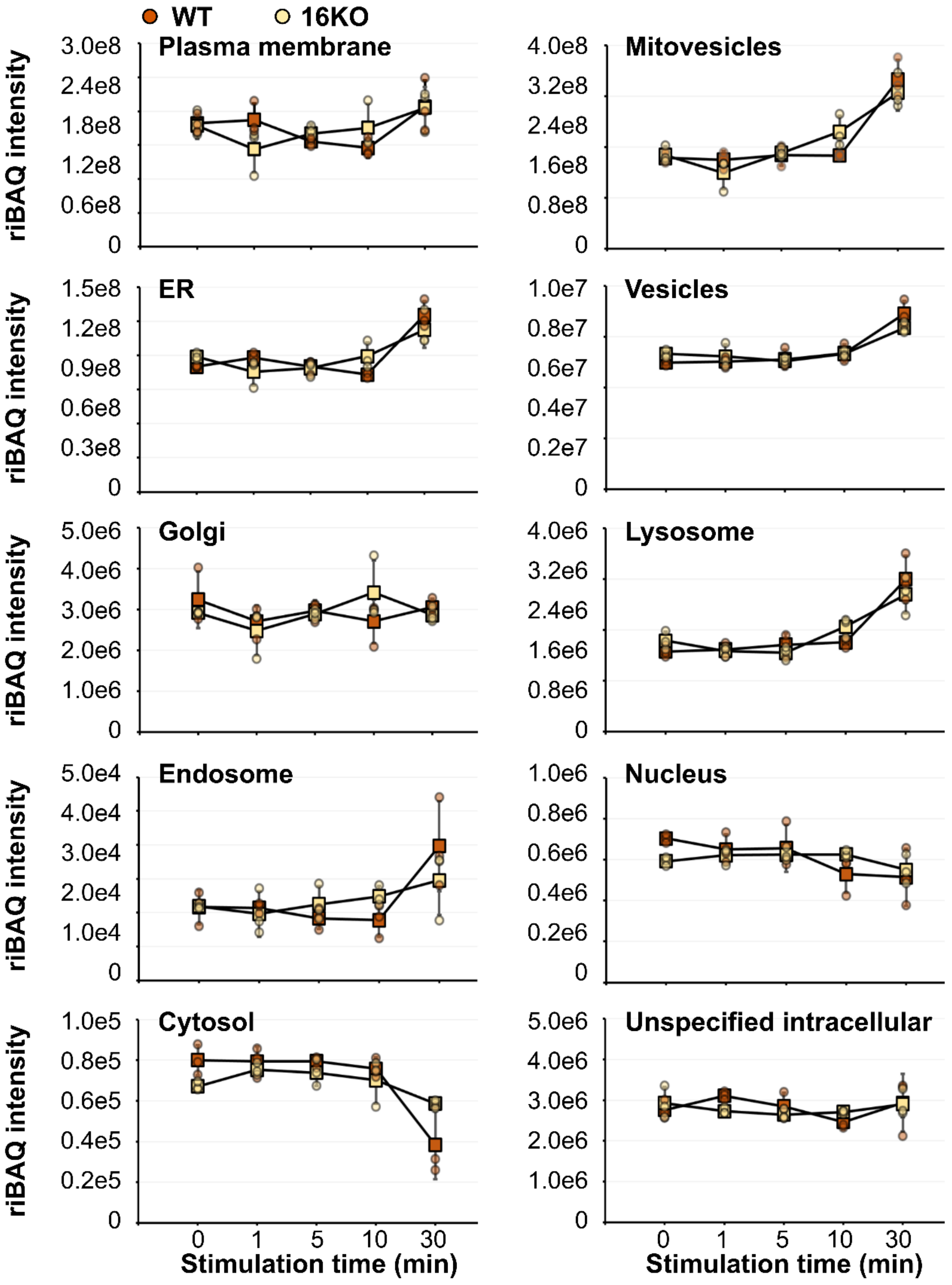

Supplementary Fig. 17 Total riBAQ intensity patterns of proteins with selected localization annotations. riBAQ intensities from all proteins with a given localization annotations were summed to obtain compartmental pattern. Mean (*dark fills*) ± standard deviation indicated, *N* = 3 (individual datapoints in *light fills*).

a

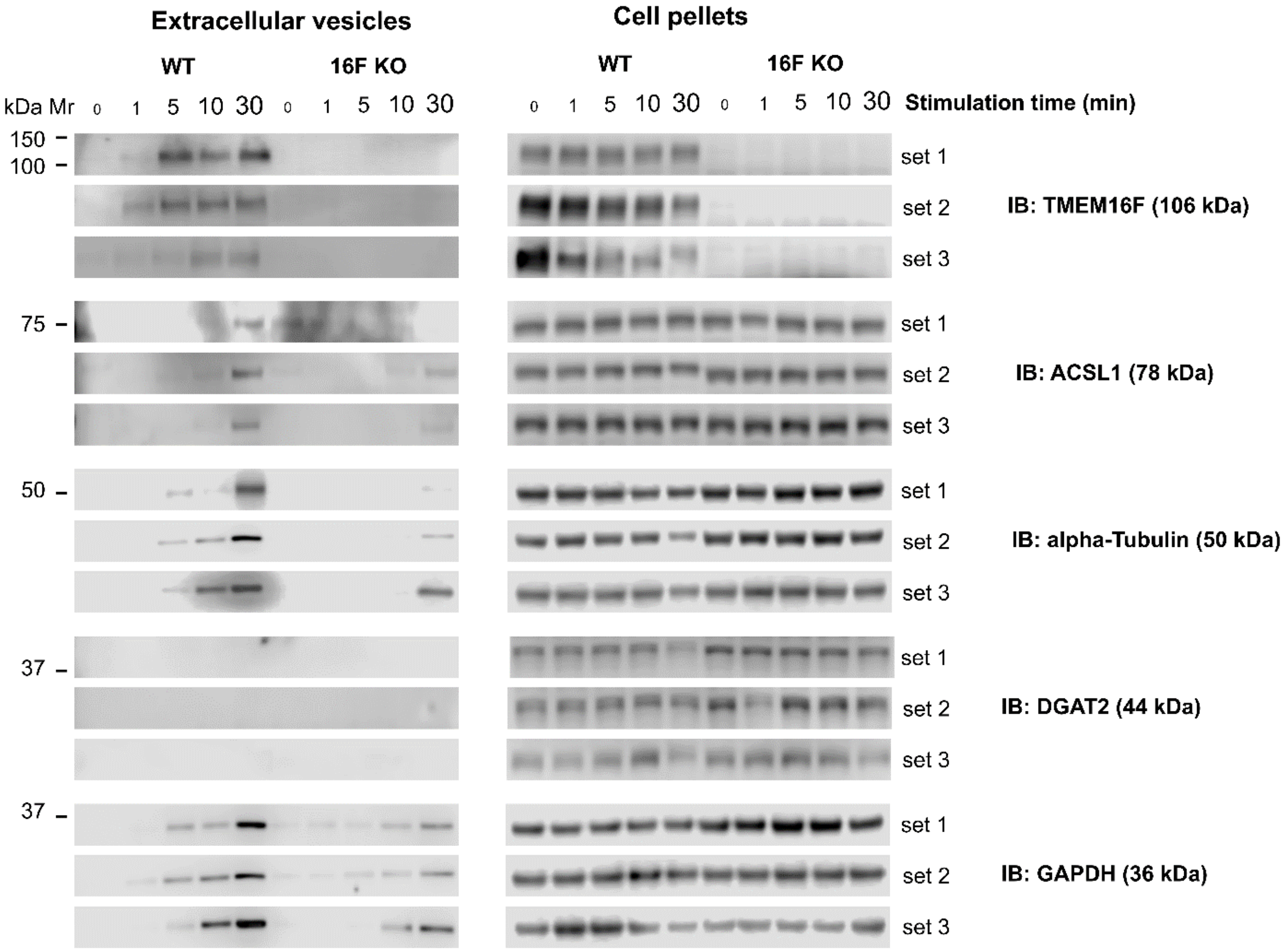
Extracellular vesicles
Cell pellets
WT
16F KO
WT
16F KO
kDa Mr
0 1 5 10 30 0 1 5 10 30
0 1 5 10 30 0 1 5 10 30
Stimulation time (min)
150
100
75
50
37
37
set 1
set 2
set 3
IB: TMEM16F (106 kDa)
IB: ACSL1 (78 kDa)
IB: alpha-Tubulin (50 kDa)
IB: DGAT2 (44 kDa)
IB: GAPDH (36 kDa)

b

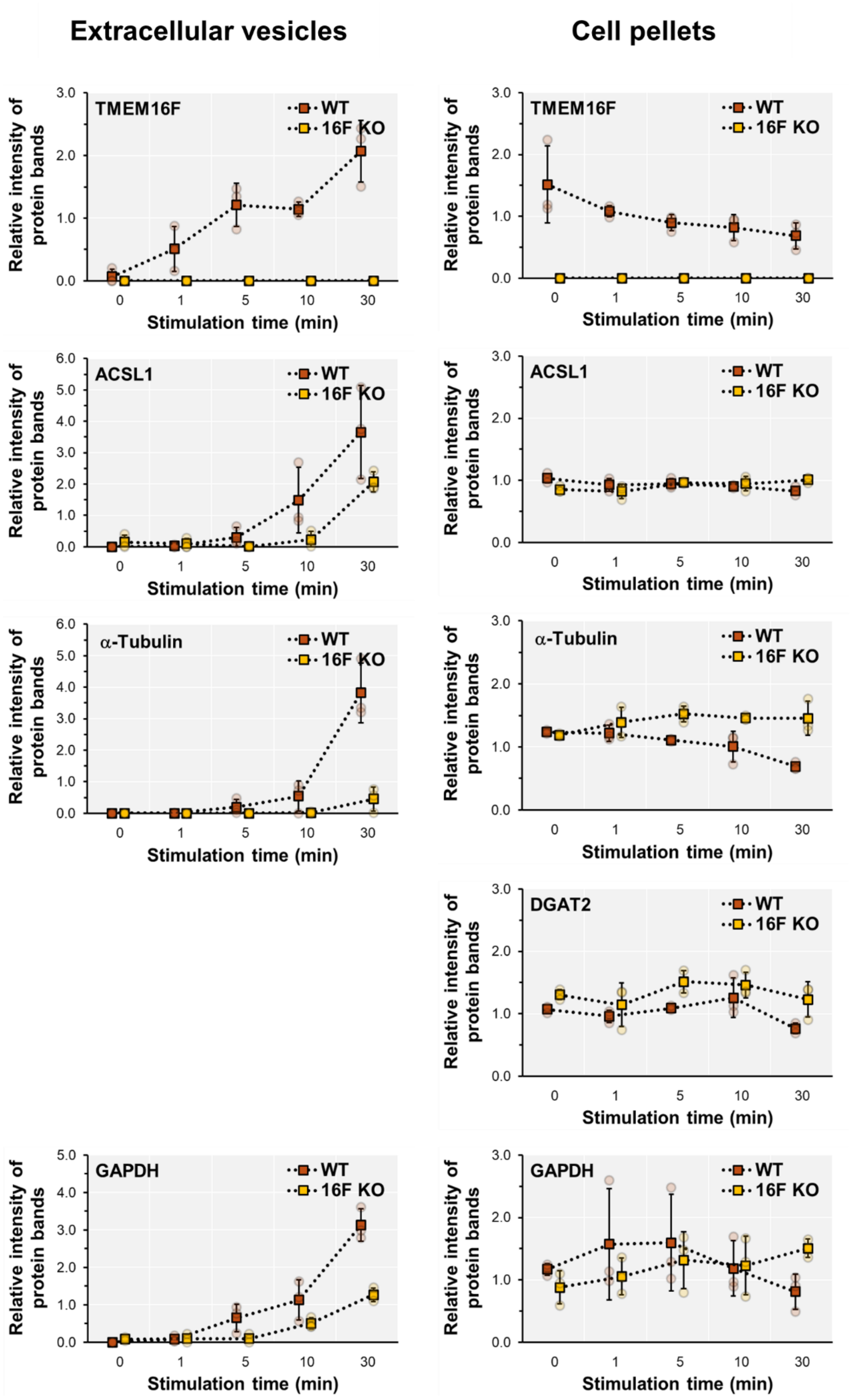
Extracellular vesicles
Cell pellets
TMEM16F
ACSL1
α-Tubulin
DGAT2
GAPDH
WT
16F KO
Relative intensity of protein bands
Stimulation time (min)
0
1
5
10
30
0.0
1.0
2.0
3.0
4.0
5.0
6.0

c

Sample:

EVs

Protein:

TMEM16F

Set:

1

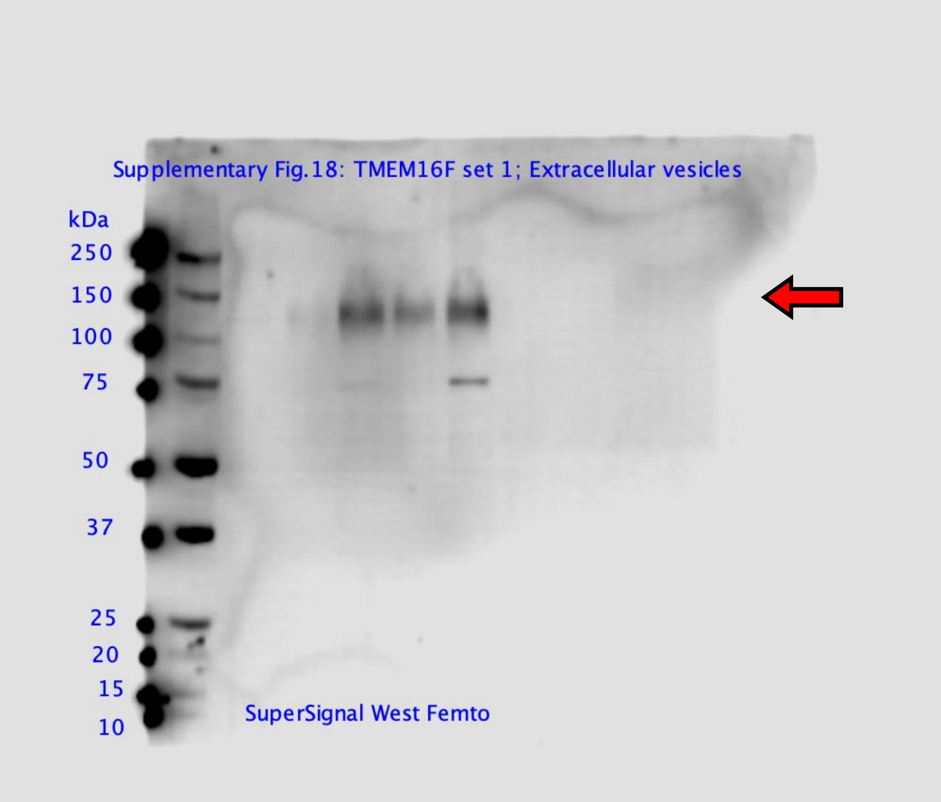

Sample:

EVs

Protein:

TMEM16F

Set:

2

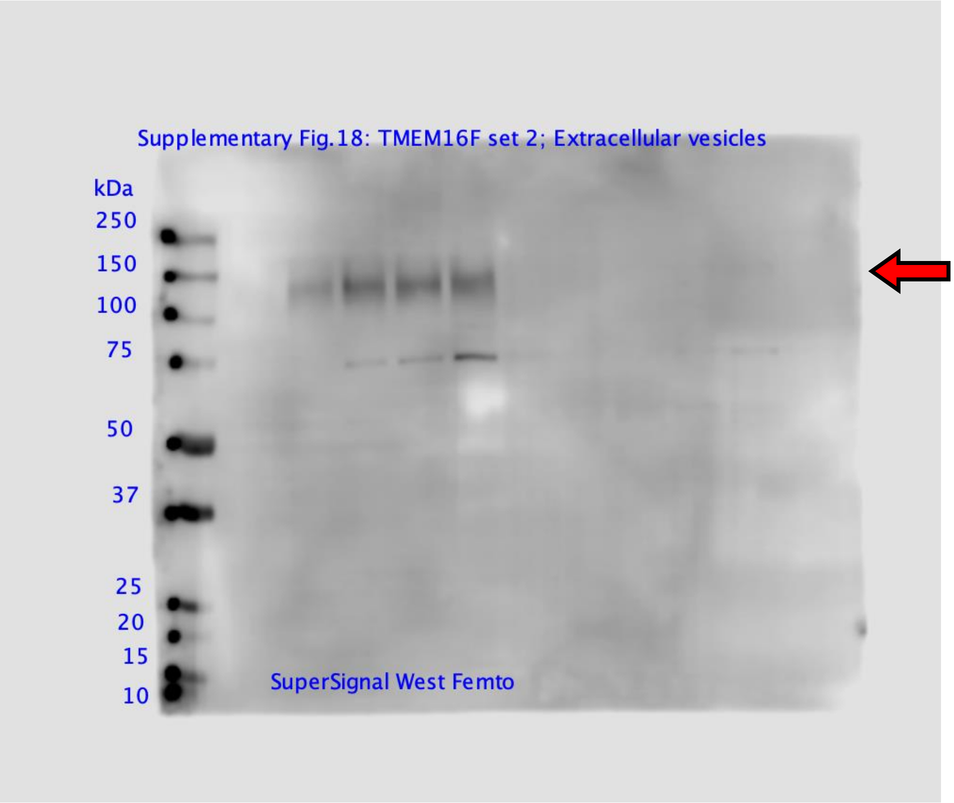

Sample:

EVs

Protein:

TMEM16F

Set:

3

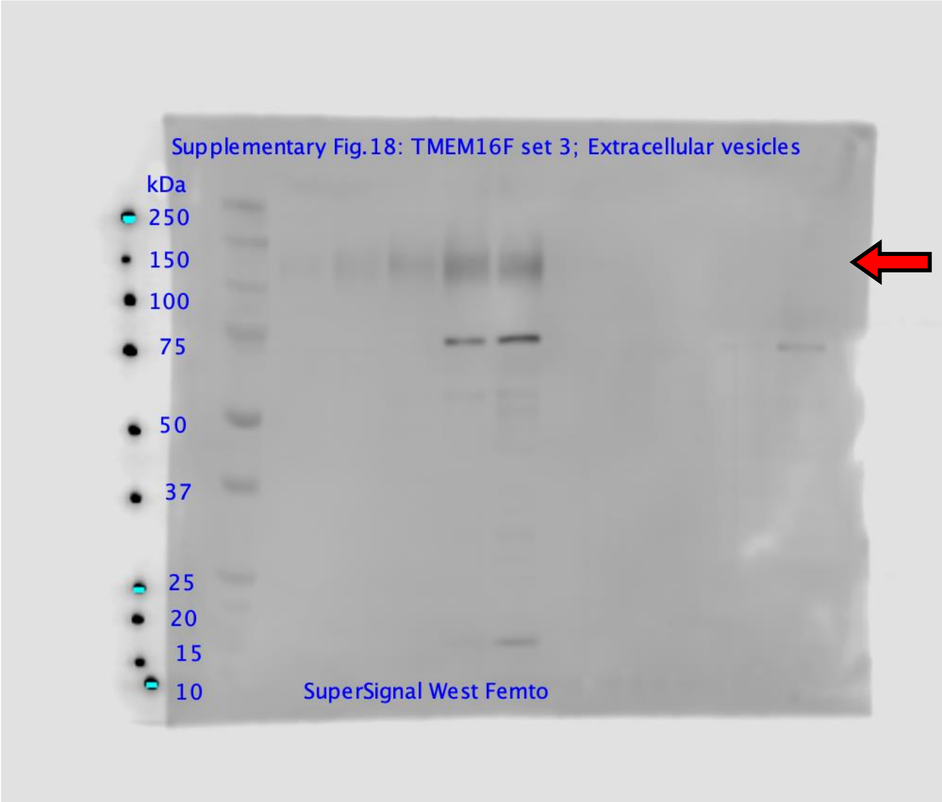

Sample:

Cell pellets

Protein:

TMEM16F

Set:

1

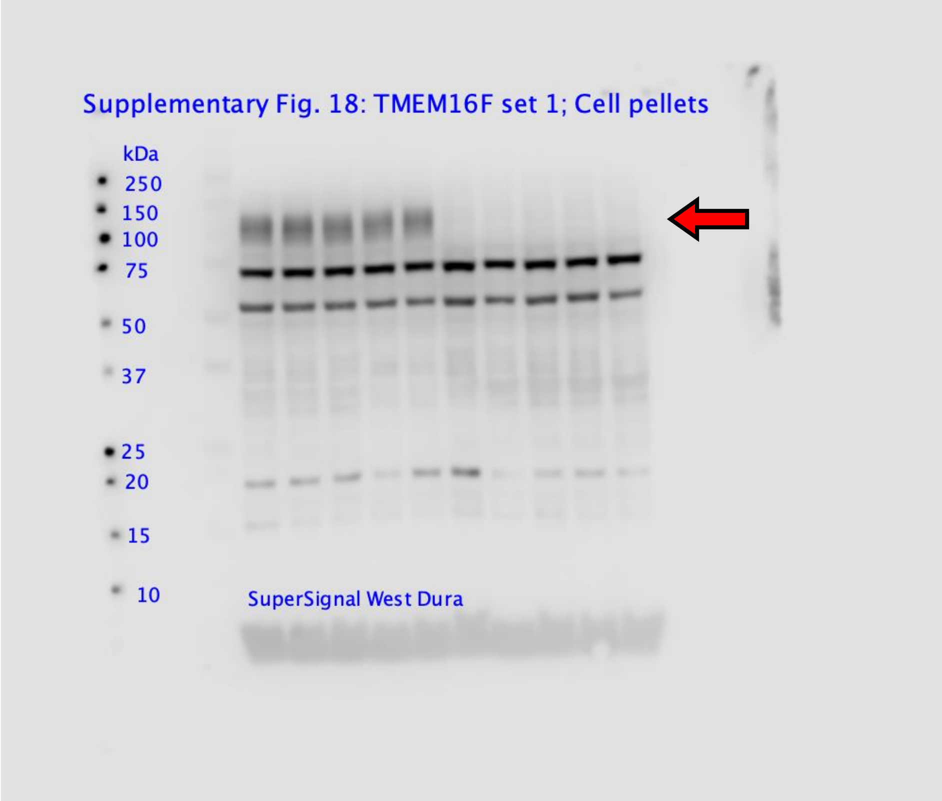

Sample:

Cell pellets

Protein:

TMEM16F

Set:

2

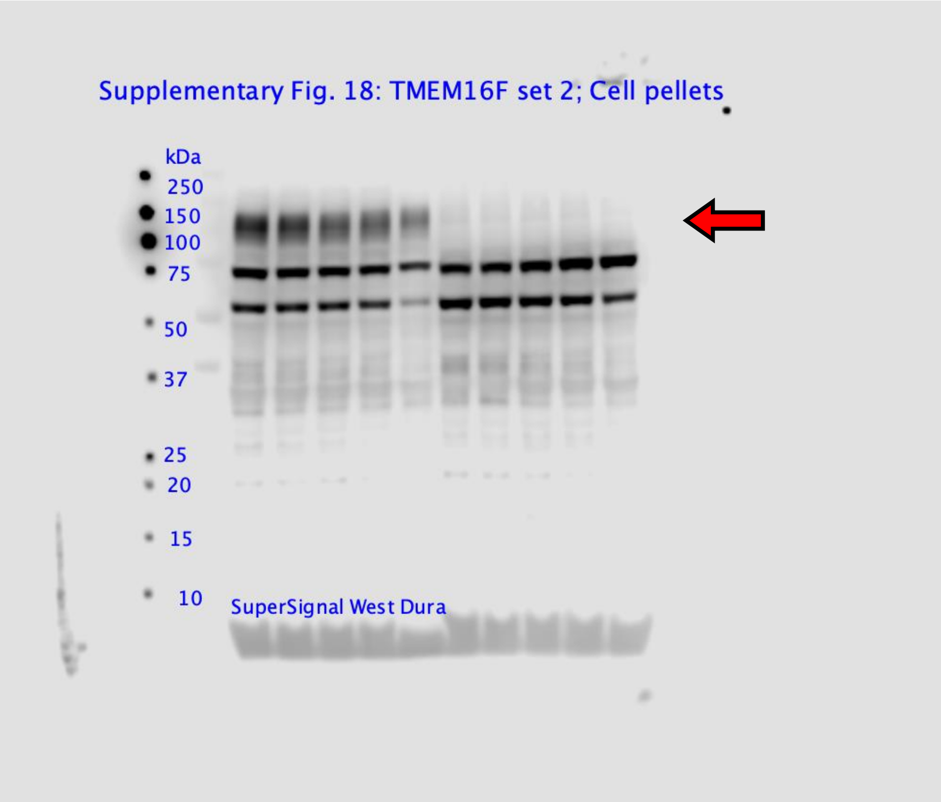

Sample:

Cell pellets

Protein:

TMEM16F

Set:

3

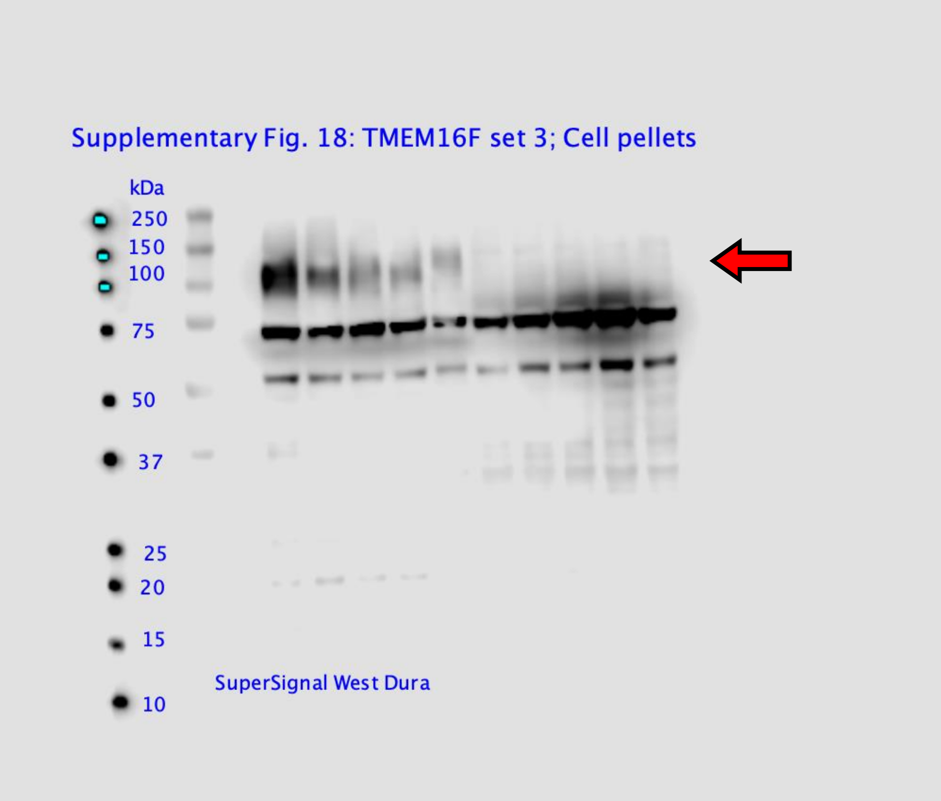

Sample:

EVs

Protein:

ACSL1

Set:

1

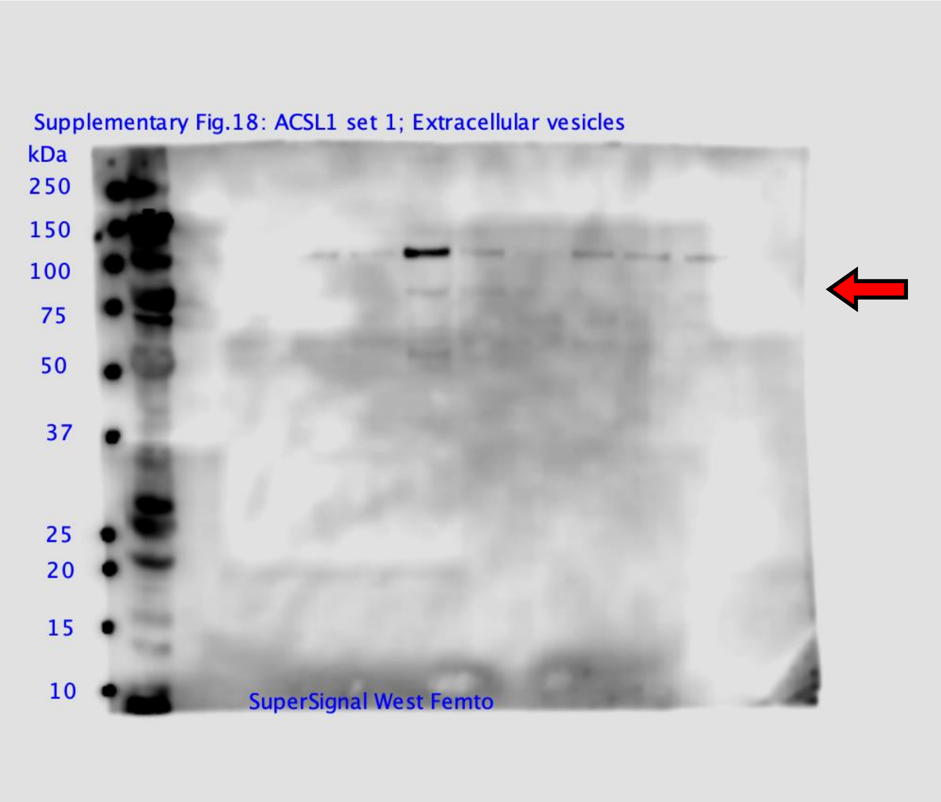

Sample:

EVs

Protein:

ACSL1

Set:

2

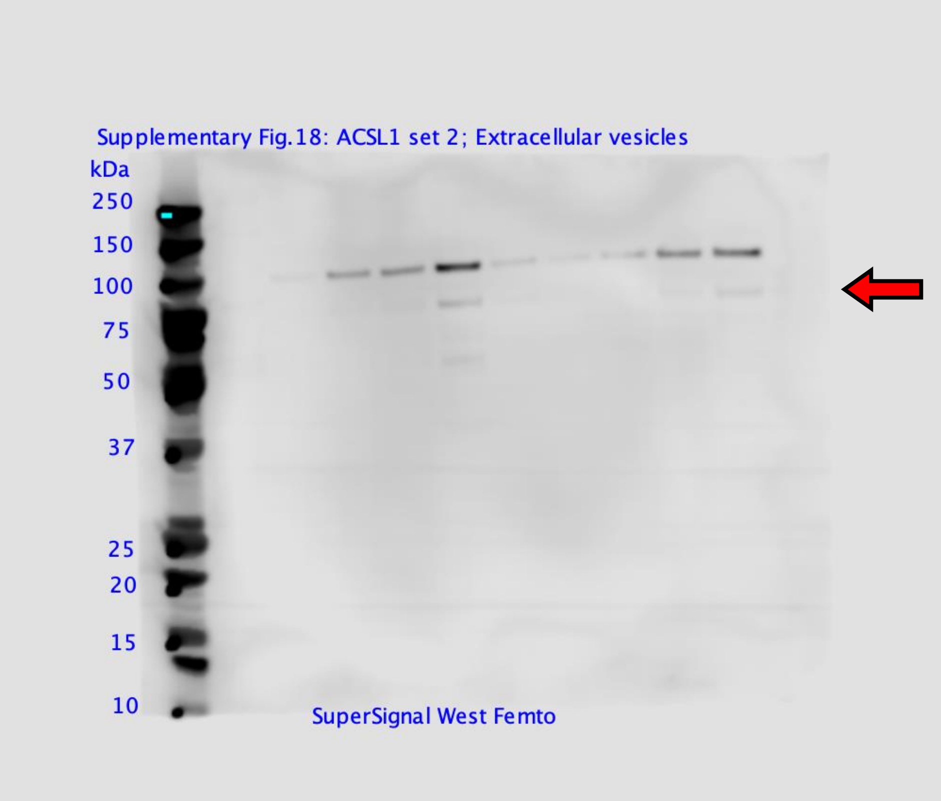

Sample:

EVs

Protein:

ACSL1

Set:

3

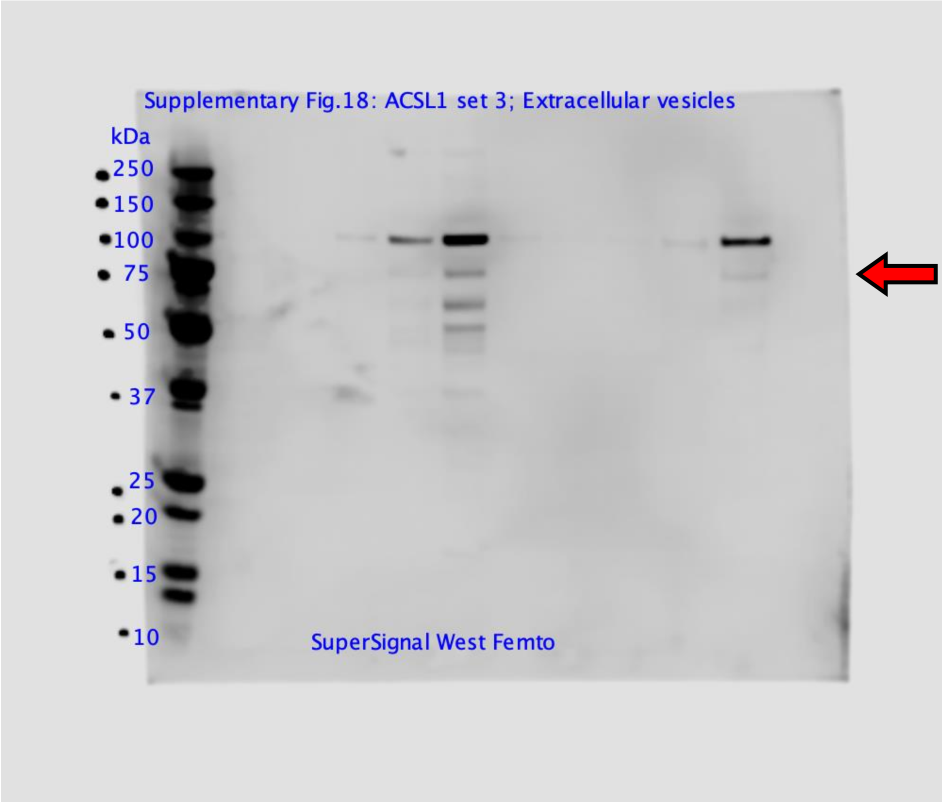

Sample:

Cell pellets

Protein:

ACSL1

Set:

1

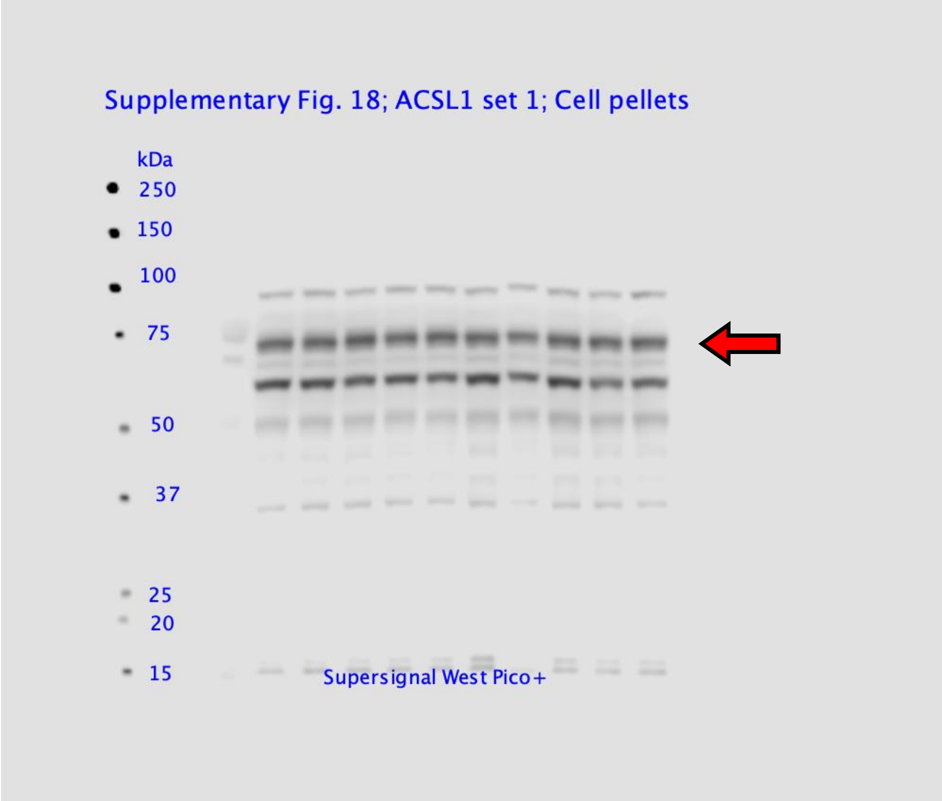

Sample:

Cell pellets

Protein:

ACSL1

Set:

2

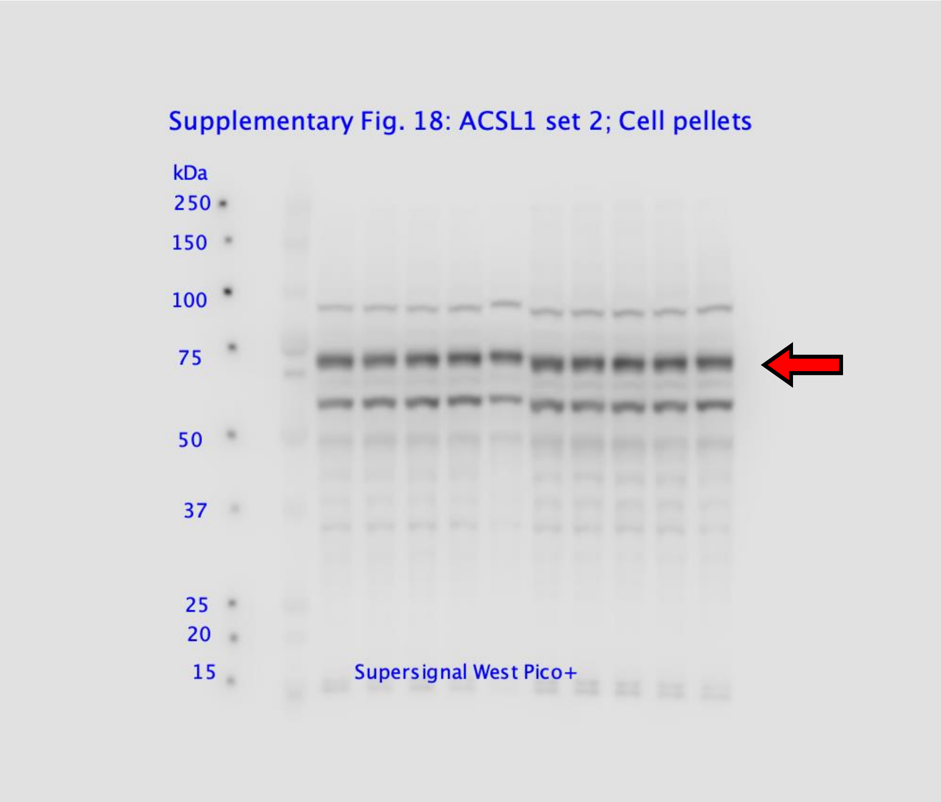

Sample:

Cell pellets

Protein:

ACSL1

Set:

3

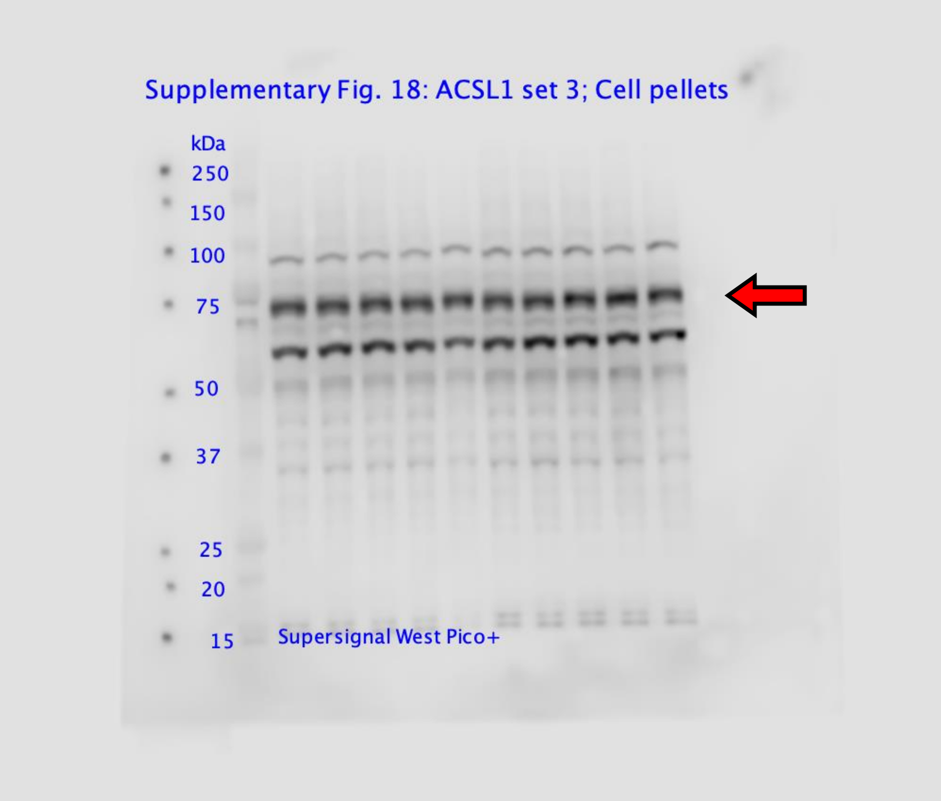

Sample:

EVs

Protein:

α-tubulin

Set:

1

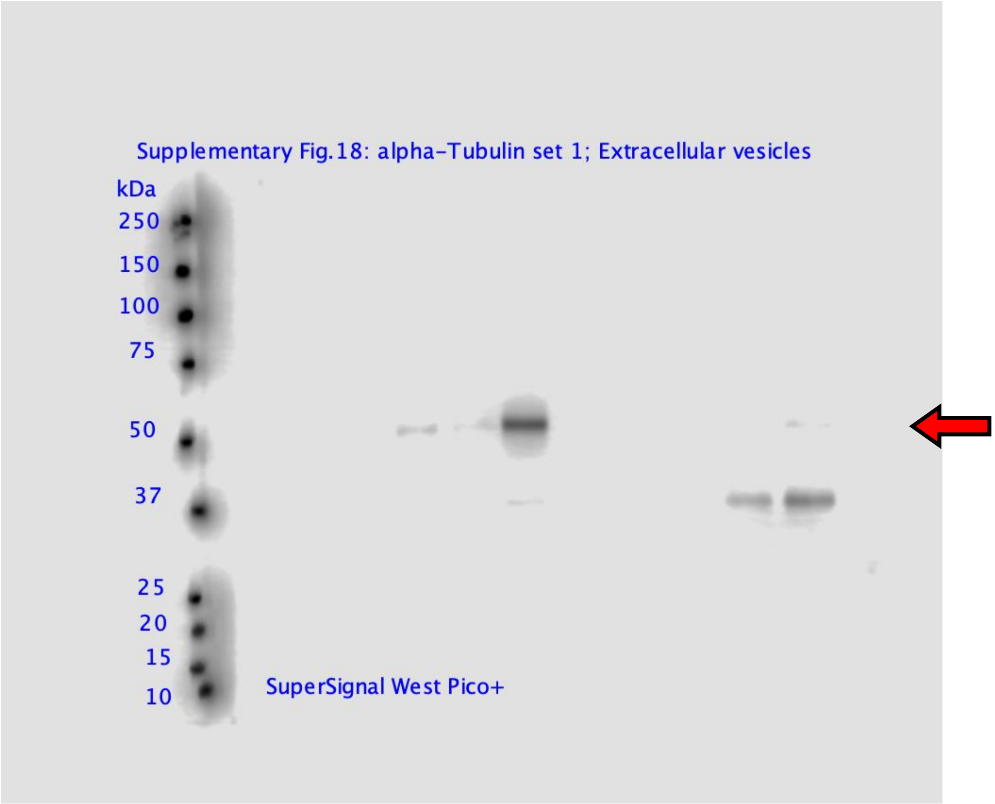

Sample:

EVs

Protein:

α-tubulin

Set:

2

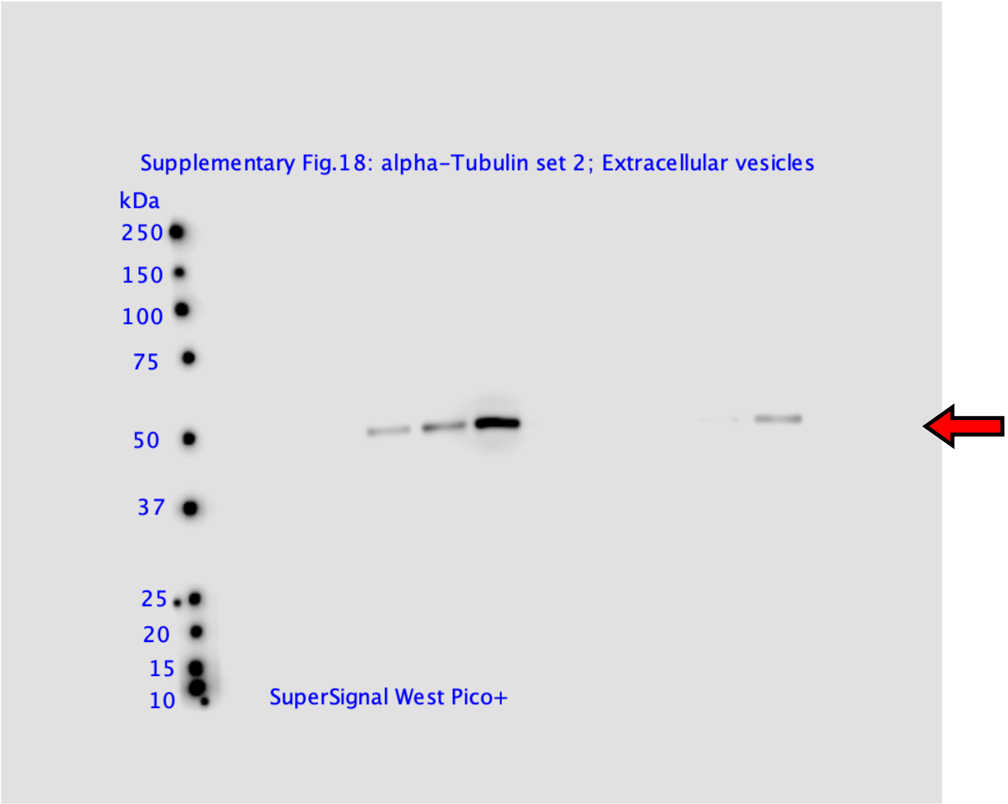

Sample:

EVs

Protein:

α-tubulin

Set:

3

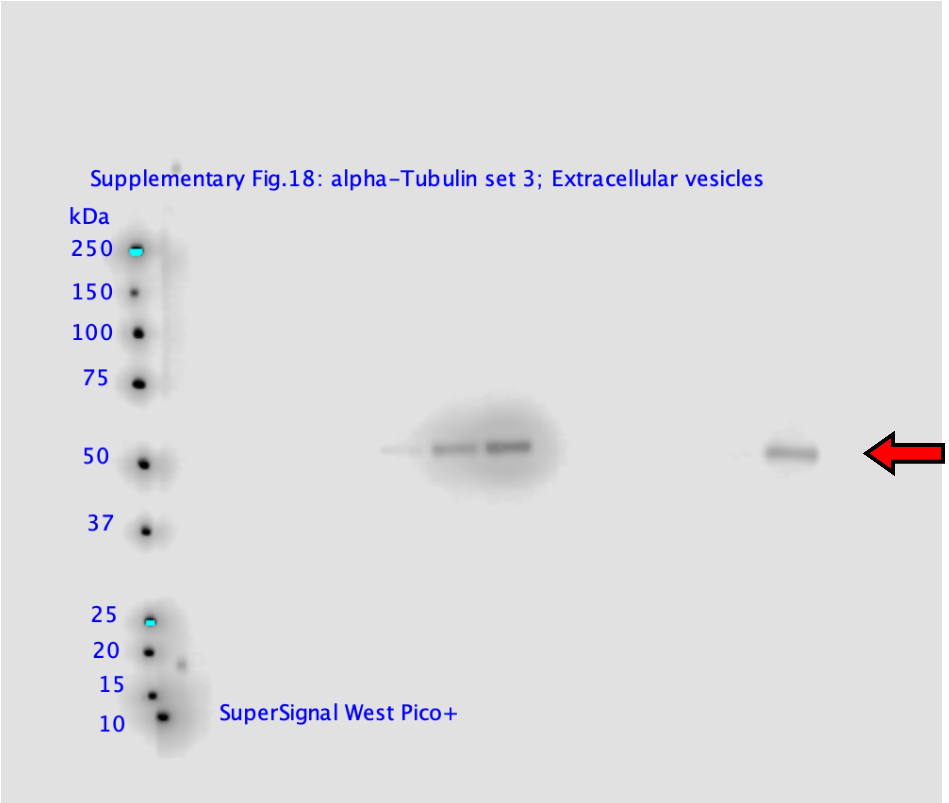

Sample:

Cell pellets

Protein:

α-tubulin

Set:

1

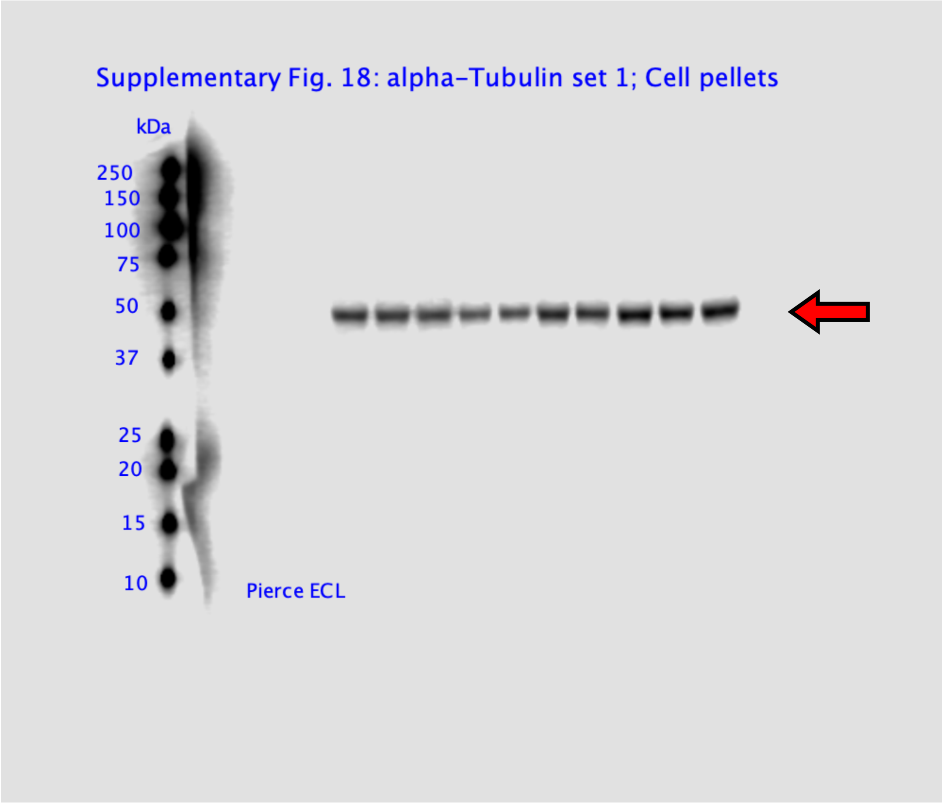

Sample:

Cell pellets

Protein:

α-tubulin

Set:

2

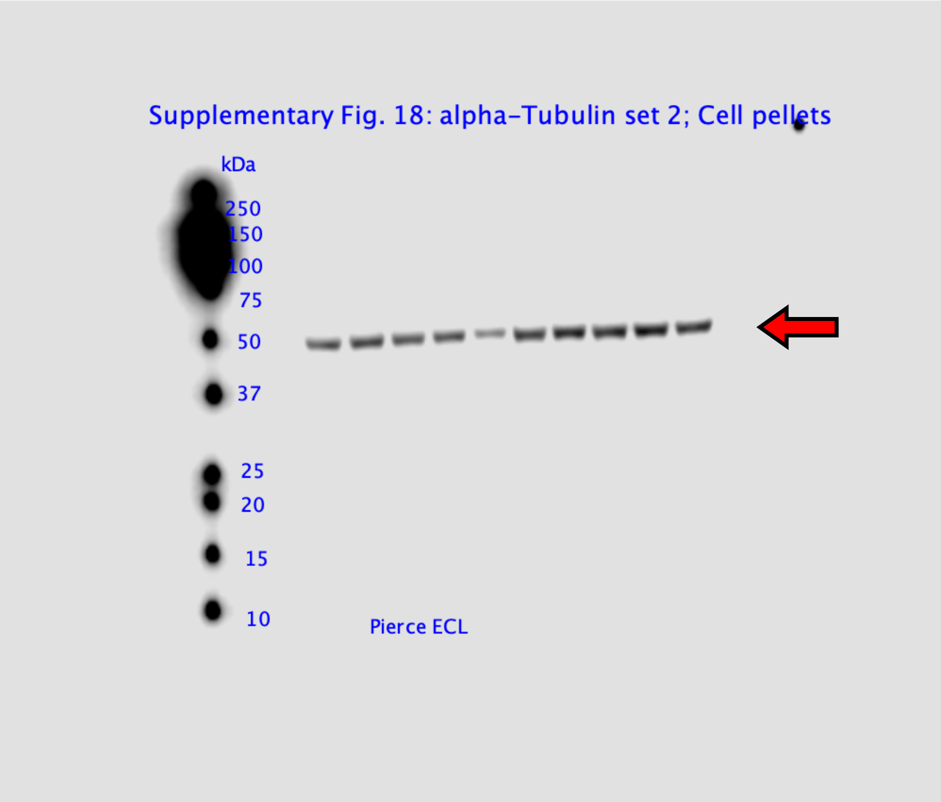

Sample:

Cell pellets

Protein:

α-tubulin

Set:

3

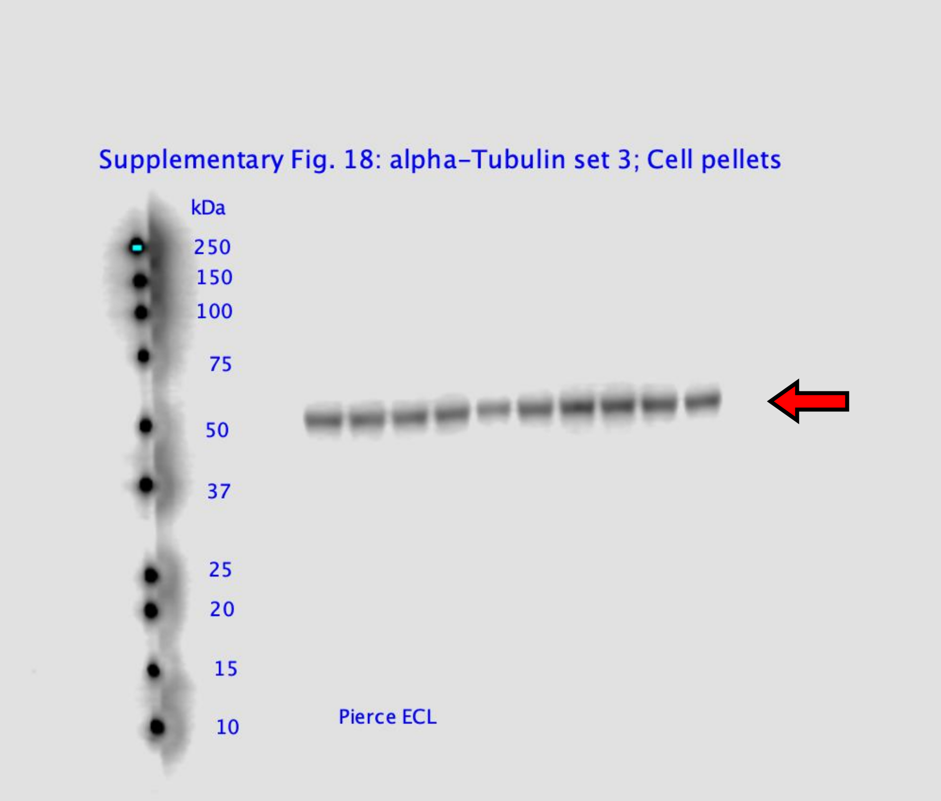

Sample:

EVs

Protein:

DGAT2

Set:

1

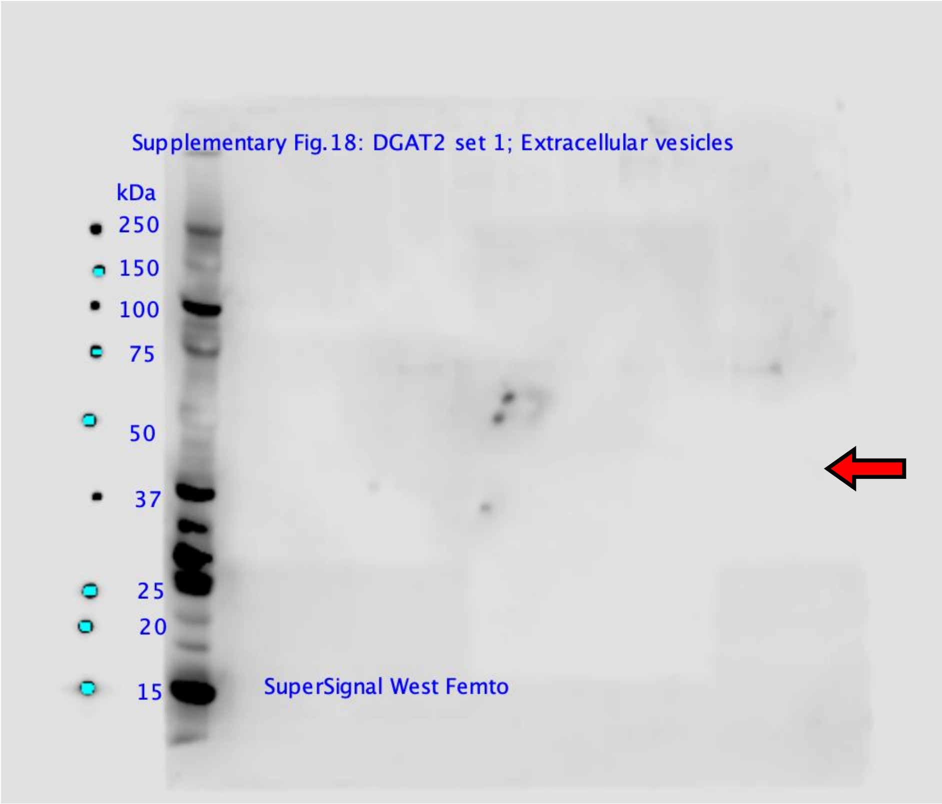

Sample:

EVs

Protein:

DGAT2

Set:

2

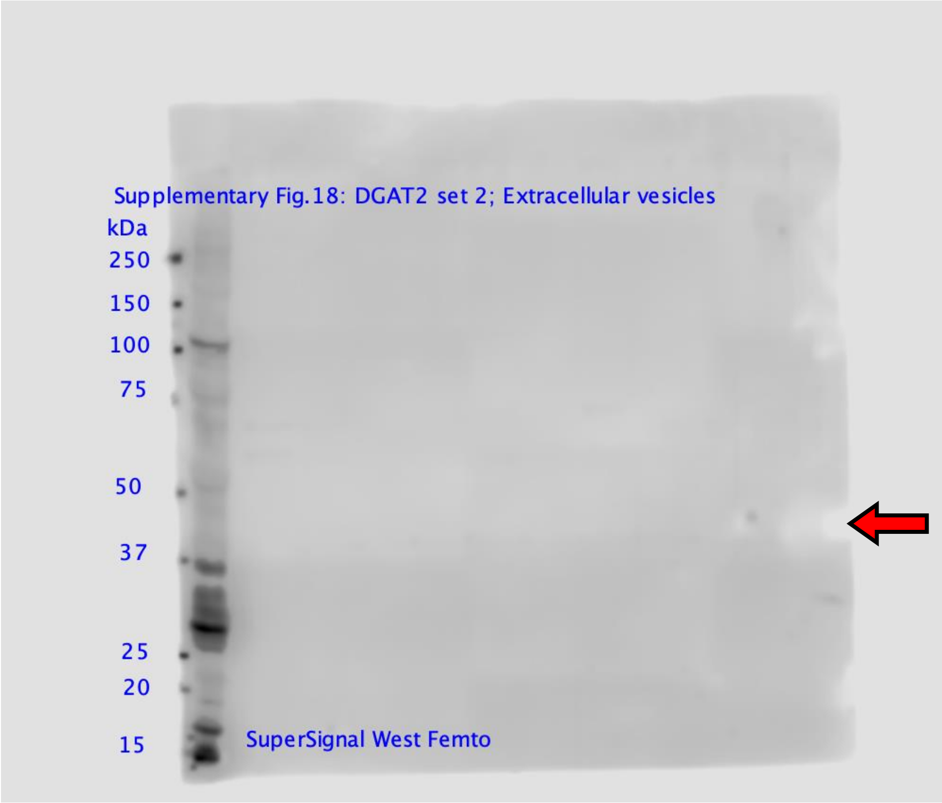

Sample:

EVs

Protein:

DGAT2

Set:

3

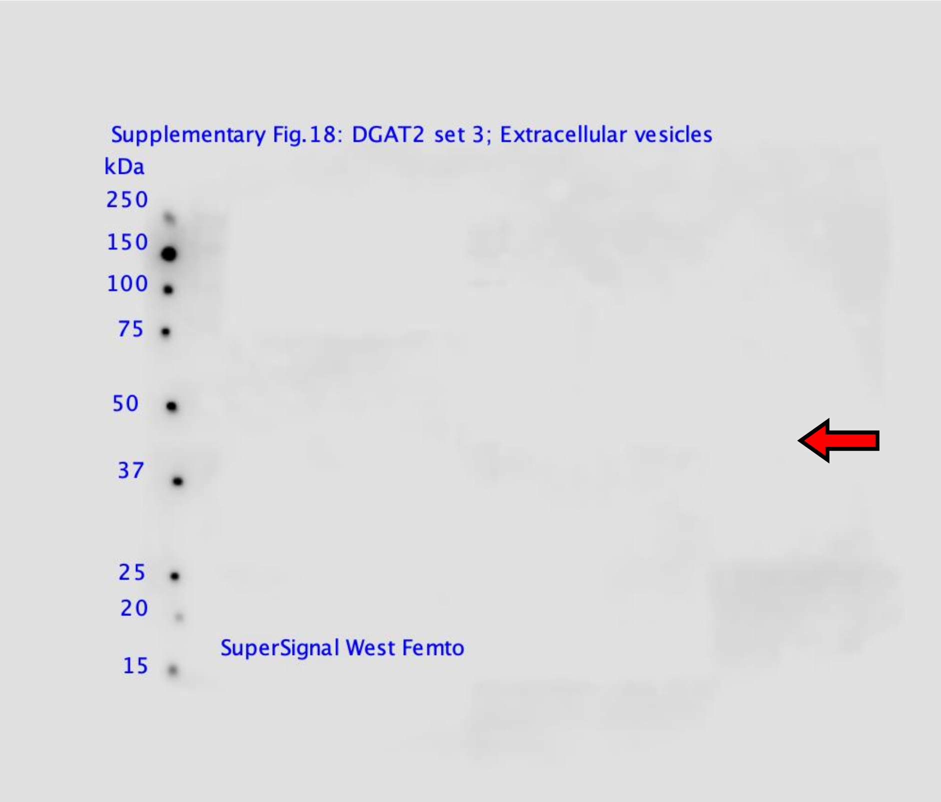

Sample:

Cell pellets

Protein:

DGAT2

Set:

1

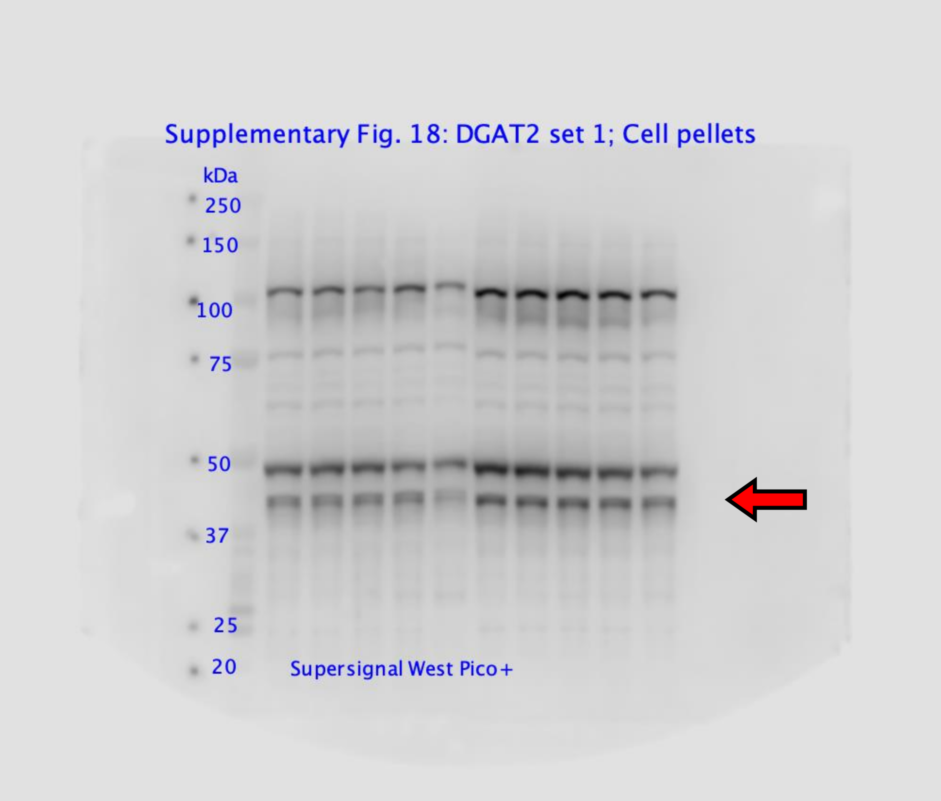

Sample:

Cell pellets

Protein:

DGAT2

Set:

2

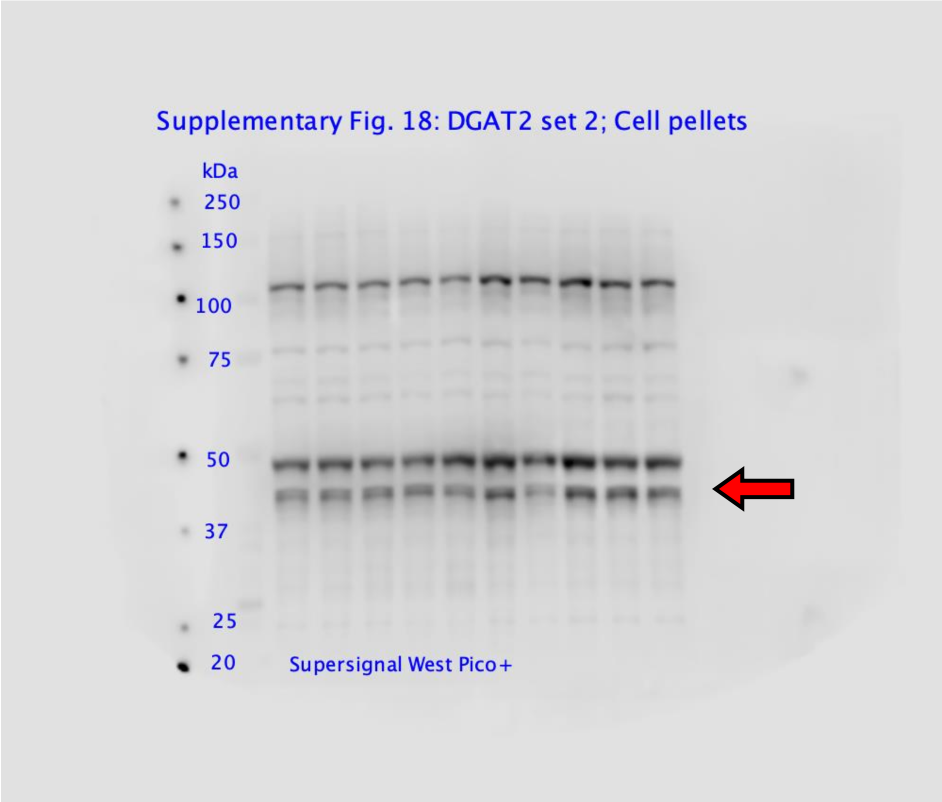

Sample:

Cell pellets

Protein:

DGAT2

Set:

3

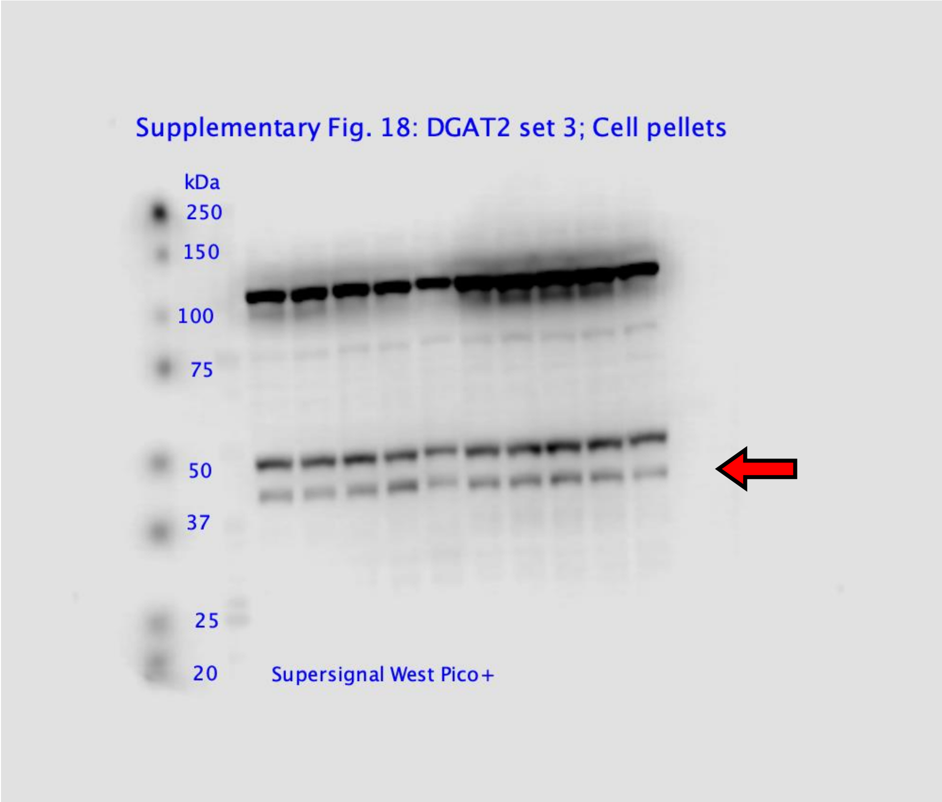

Sample:

EVs

Protein:

GAPDH

Set:

1

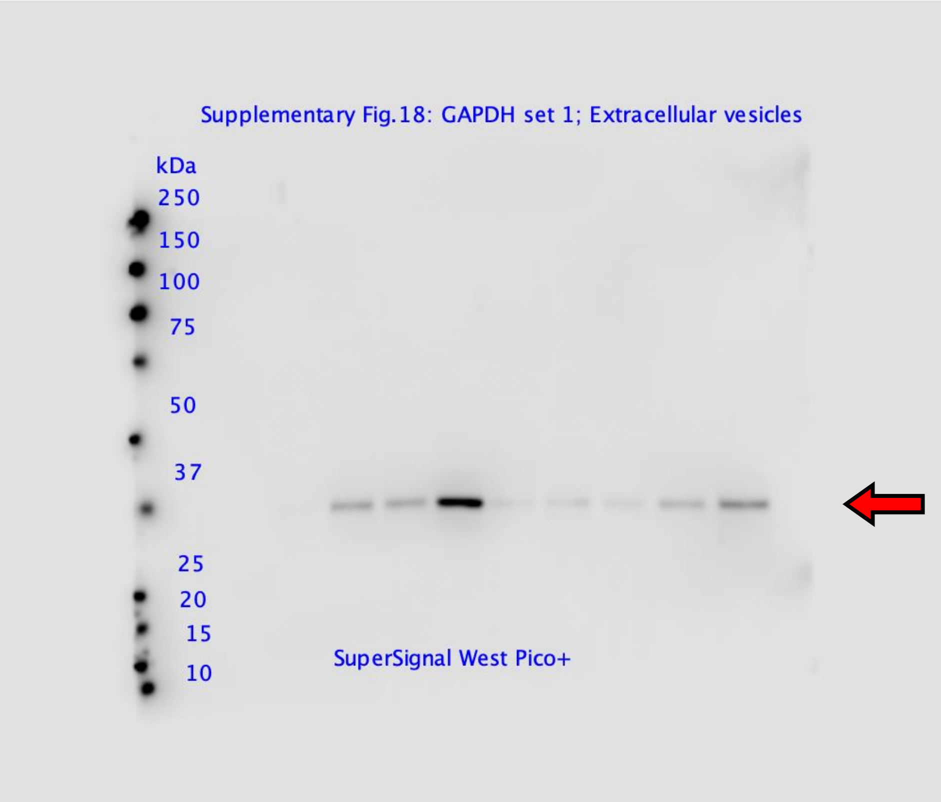

Sample:

EVs

Protein:

GAPDH

Set:

2

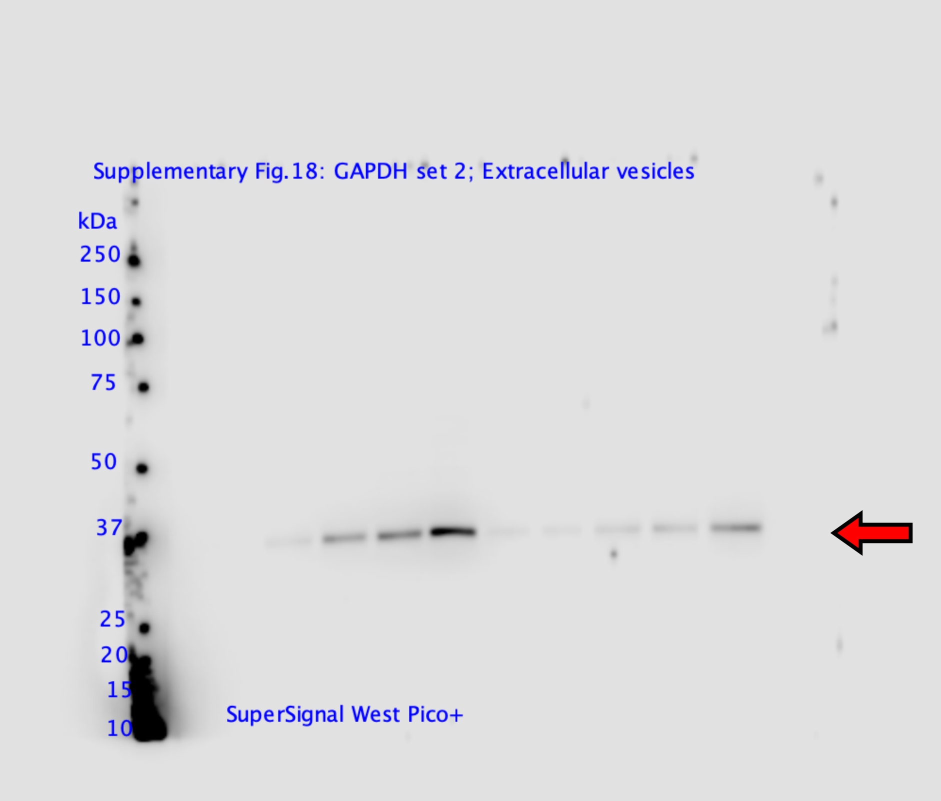

Sample:

EVs

Protein:

GAPDH

Set:

3

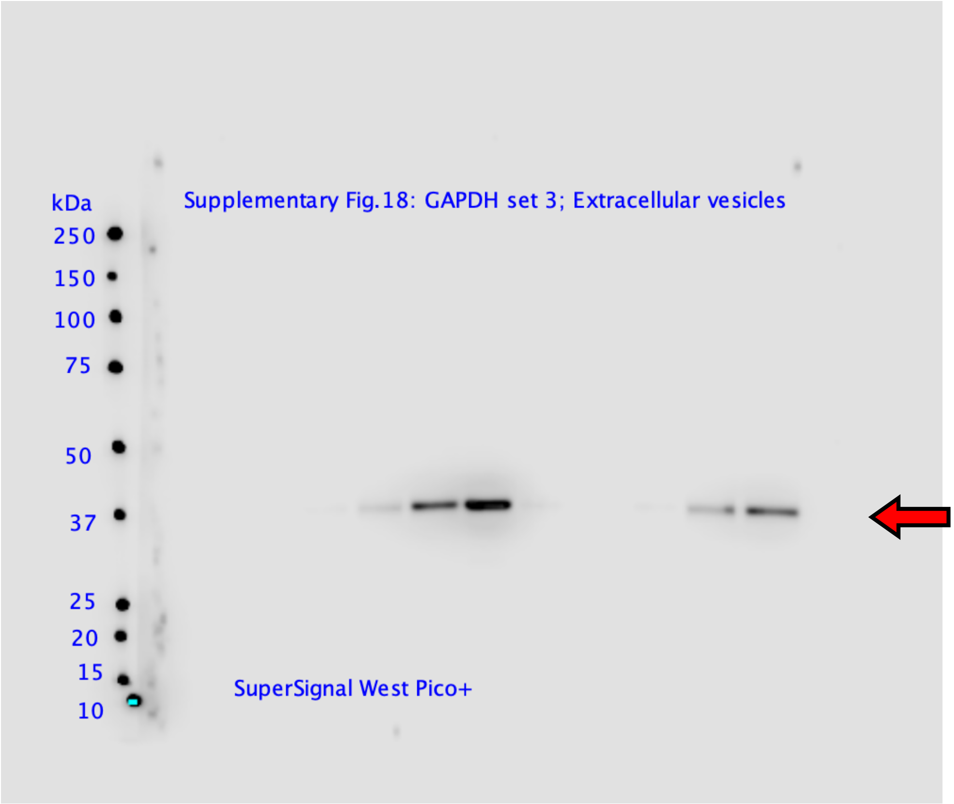

Sample:

Cell pellets

Protein:

GAPDH

Set:

1

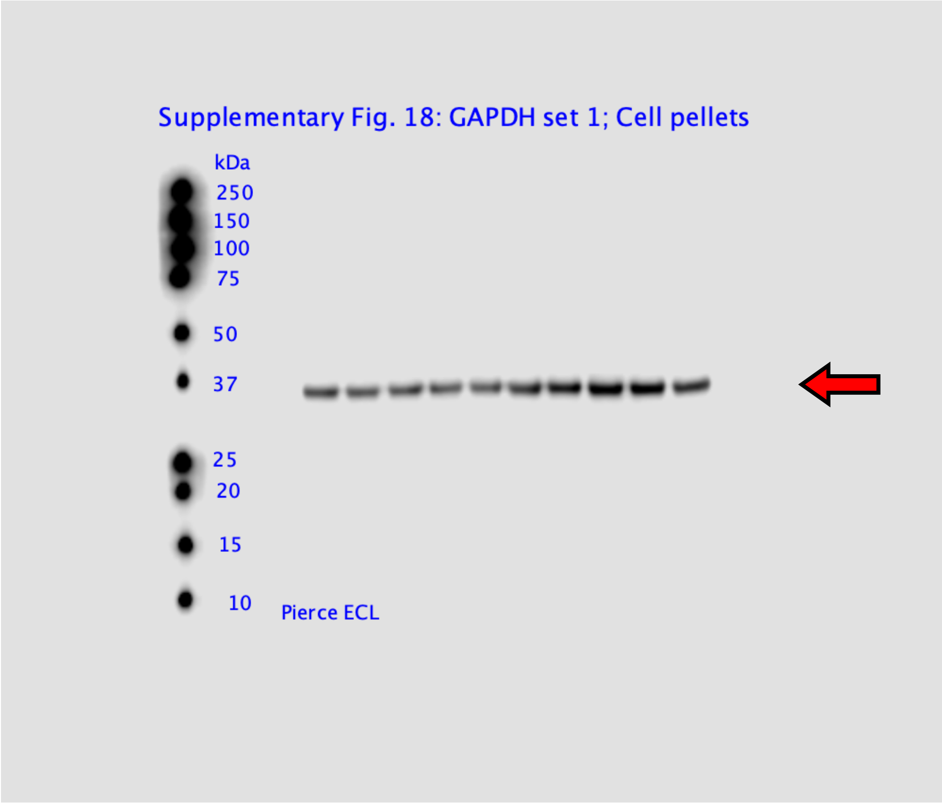

Sample:

Cell pellets

Protein:

GAPDH

Set:

2

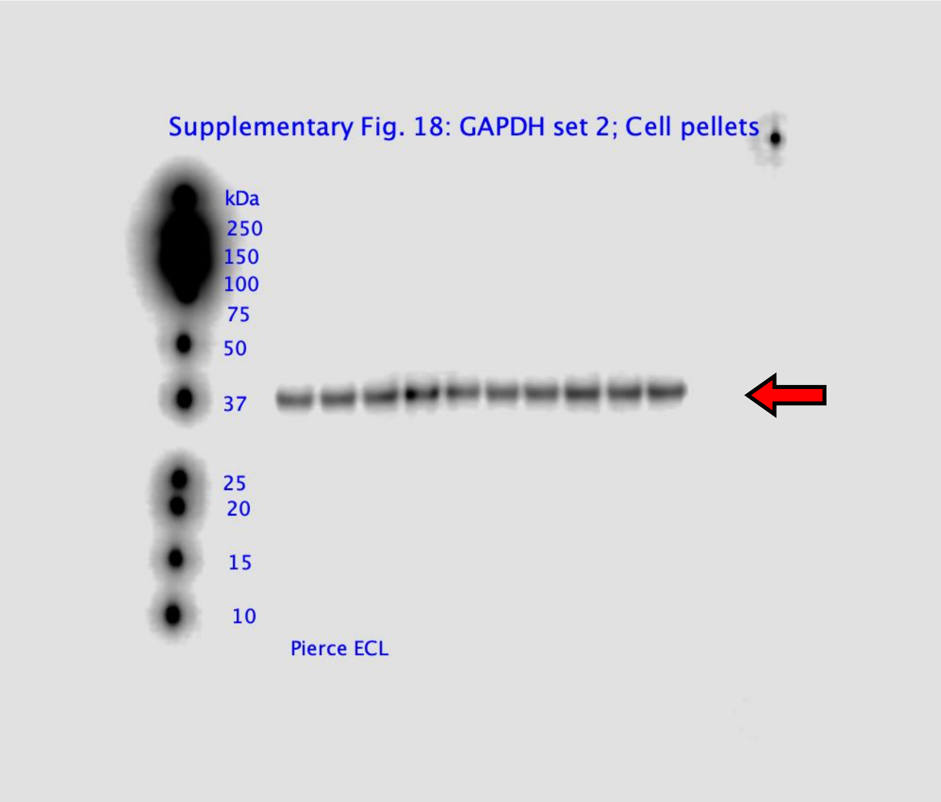

Sample:

Cell pellets

Protein:

GAPDH

Set:

3

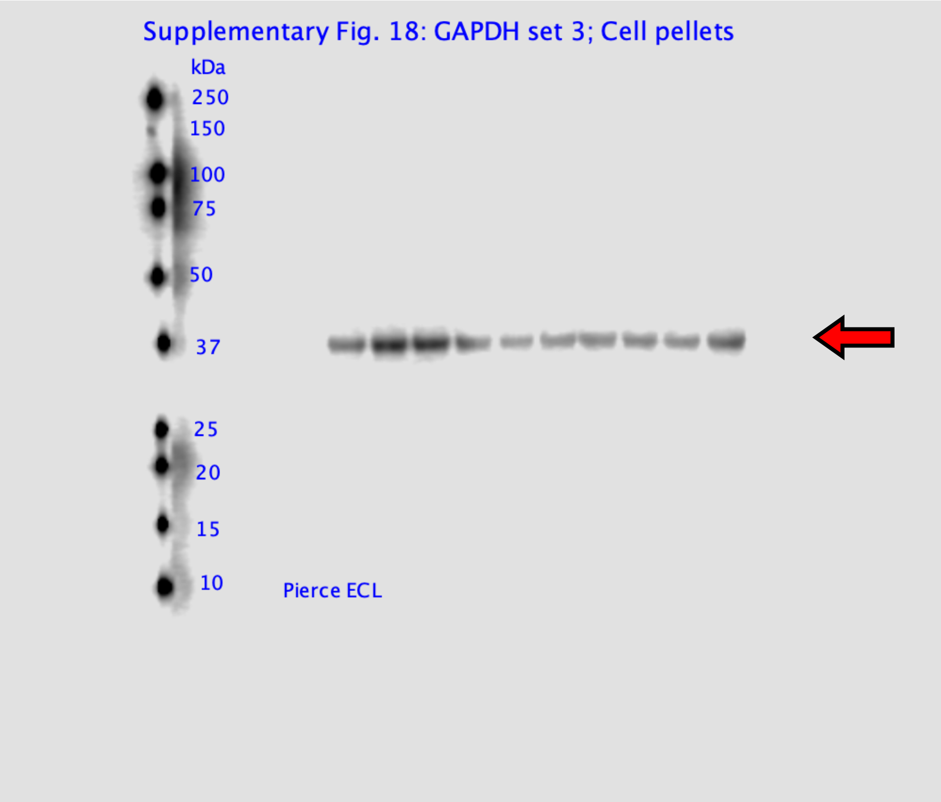

Supplementary Fig. 18 ***(17 previous pages)*** **Western blotting of extracellular vesicle and cell pellet fractions from WT and 16FKO Jurkat cells at various time points after ionomycin stimulation.** Antibodies against TMEM16F, ACSL1, α-tubulin, DGAT2, and GAPDH proteins were used for protein detection. **a** Cropped western blots with the relevant regions shown (see c for uncropped blots). Data from three independent replicates are shown. **b** Densitometric quantitation of the protein bands. Mean (*dark fills*) ± standard deviation indicated, $N = 3$ (individual datapoints in *light fills*). The densitometric data were normalized for comparable replicates. **c** Uncropped western blots. Red arrow indicates the approximate molecular weight of the protein of interest. Mr, relative molecular weight; kDa, kilodalton.
