# Supplementary material for: SLAPSHOT reveals rapid dynamics of extracellularly exposed proteome in response to calcium-activated plasma membrane phospholipid scrambling
Source: Commun Biol. 2024 Aug 29;7:1060. doi: 10.1038/s42003-024-06729-x (PMC11362511; doi:10.1038/s42003-024-06729-x)
Supplement: Supplementary file 3 — Description of Additional Supplementary File [file 42003_2024_6729_MOESM3_ESM.pdf]

Description of Additional Supplementary Files

File name: Supplementary Data 1

Description: Supplementary Tables and Supporting Data for figures.

File name: Supplementary Data 2

Description: Supporting R code
